# Supplementary material for: Autonomous language-image generation loops converge to generic visual motifs
Source: Patterns (N Y). 2025 Dec 19;7(1):101451. doi: 10.1016/j.patter.2025.101451 (PMC12827715; doi:10.1016/j.patter.2025.101451)
Supplement: Document S1. Figures S1–S17 and Methods S1 and S2 [file mmc1.pdf]

Patterns, Volume *7*

Supplemental information

Autonomous language-image generation loops converge to generic visual motifs

Arend Hintze, Frida Proschinger Åström, and Jory Schossau

Supplemental Methods 1

October 26, 2025

Converged Image Mosaics After 100 Iterations

The following figures (S1–S16) show the final-step image mosaics from each of the 16 generator–describer model combinations. Each mosaic illustrates the end point of semantic drift, revealing visually similar but subtly model-specific convergences.

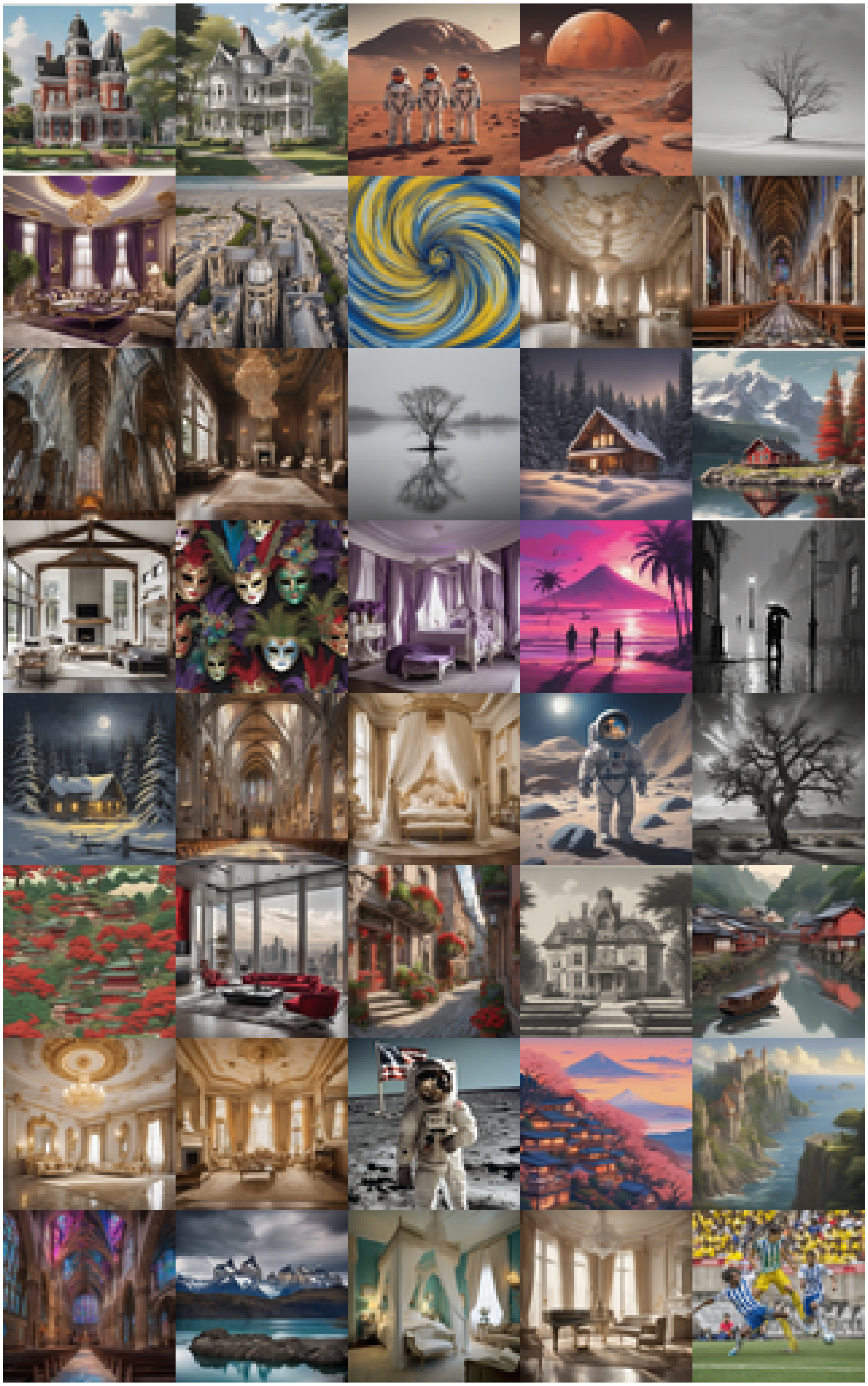

Figure S1: Generator: `stable-diffusion-xl-base-1.0`, Describer: `llava`.

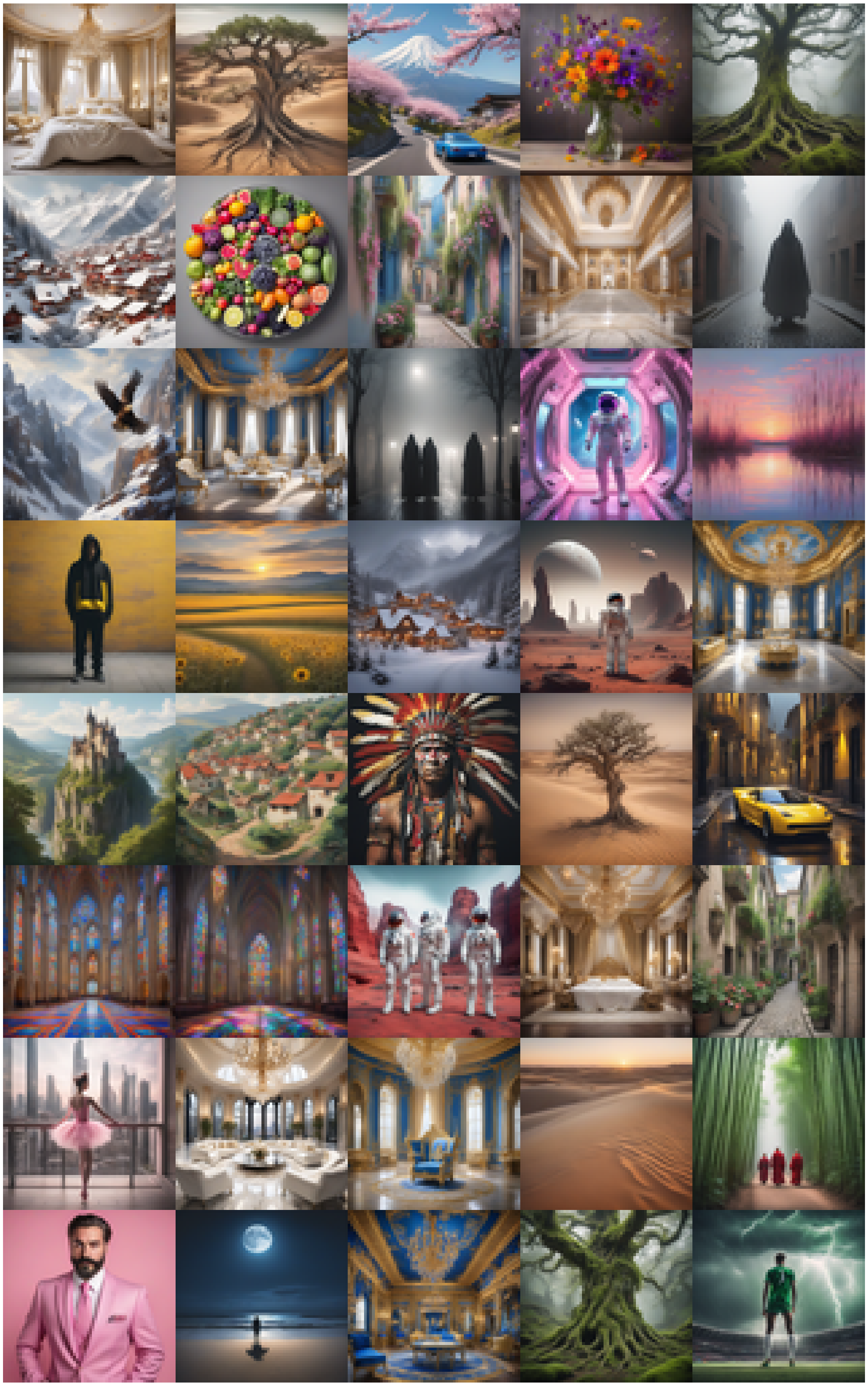

Figure S2: Generator: `segmind-SSD-1B`, Describer: `llava`.

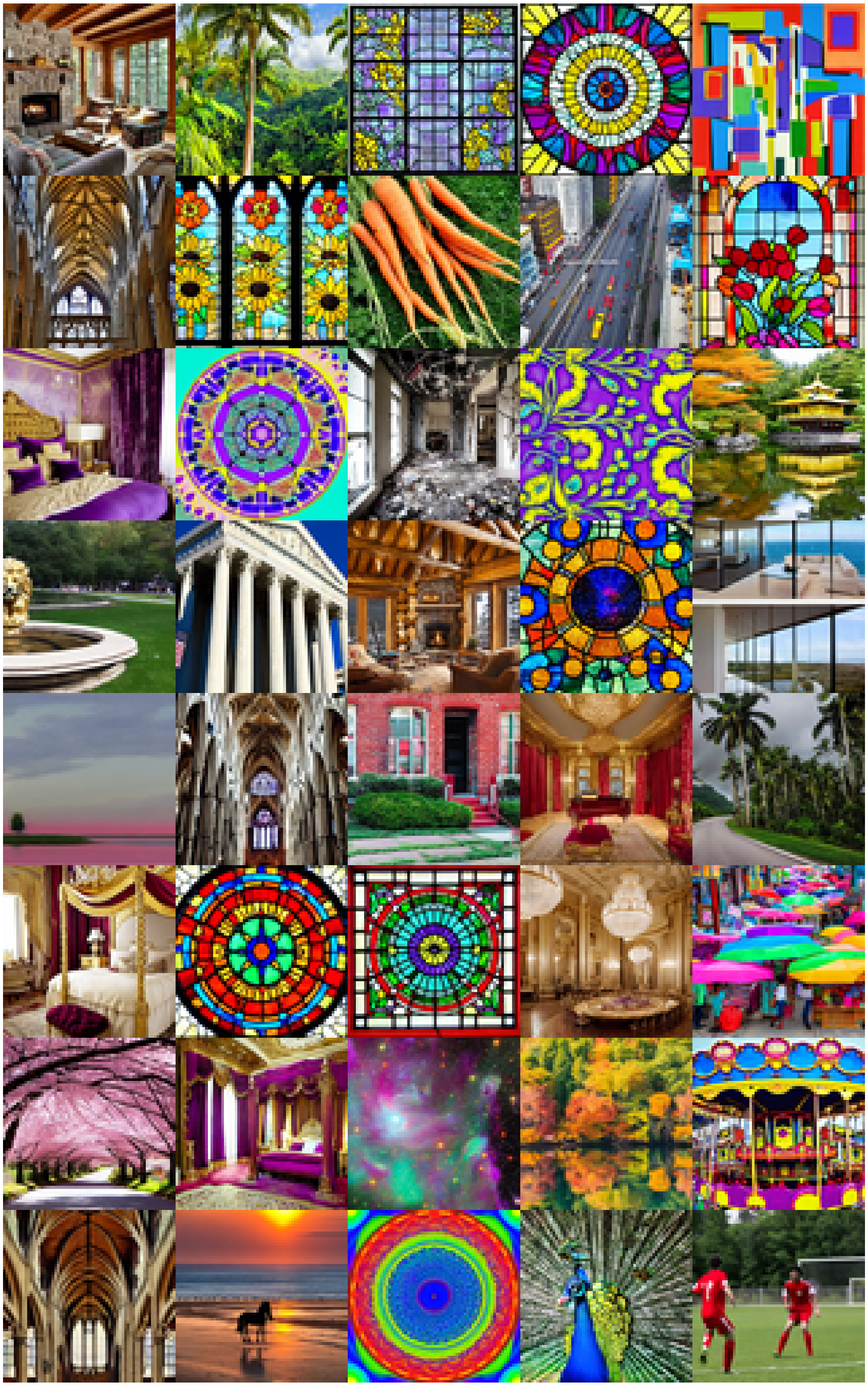

Figure S3: Generator: `stable-diffusion-v1.5`, Describer: `llava`.

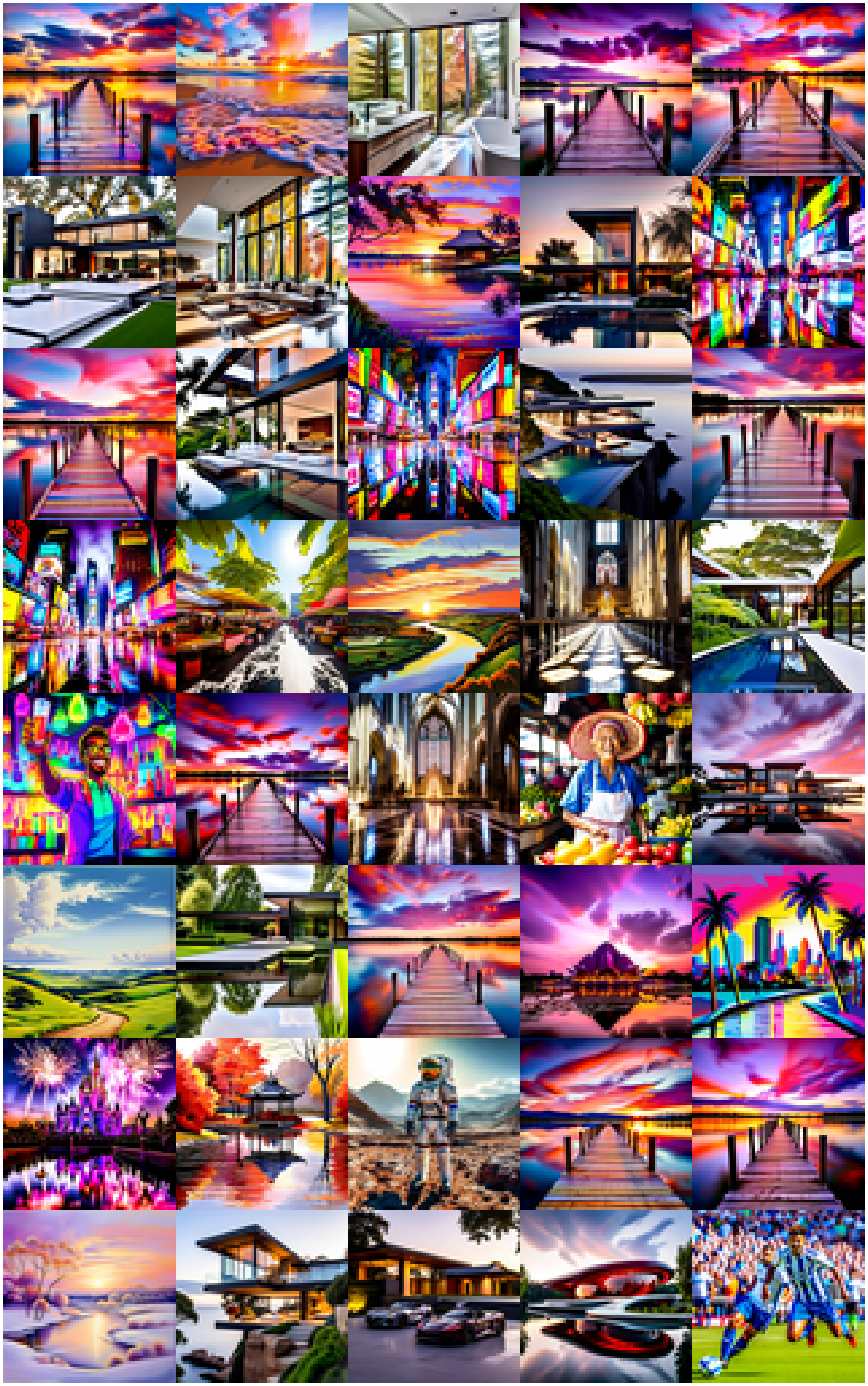

Figure S4: Generator: `playground-v2-aesthetic`, Describer: `llava`.

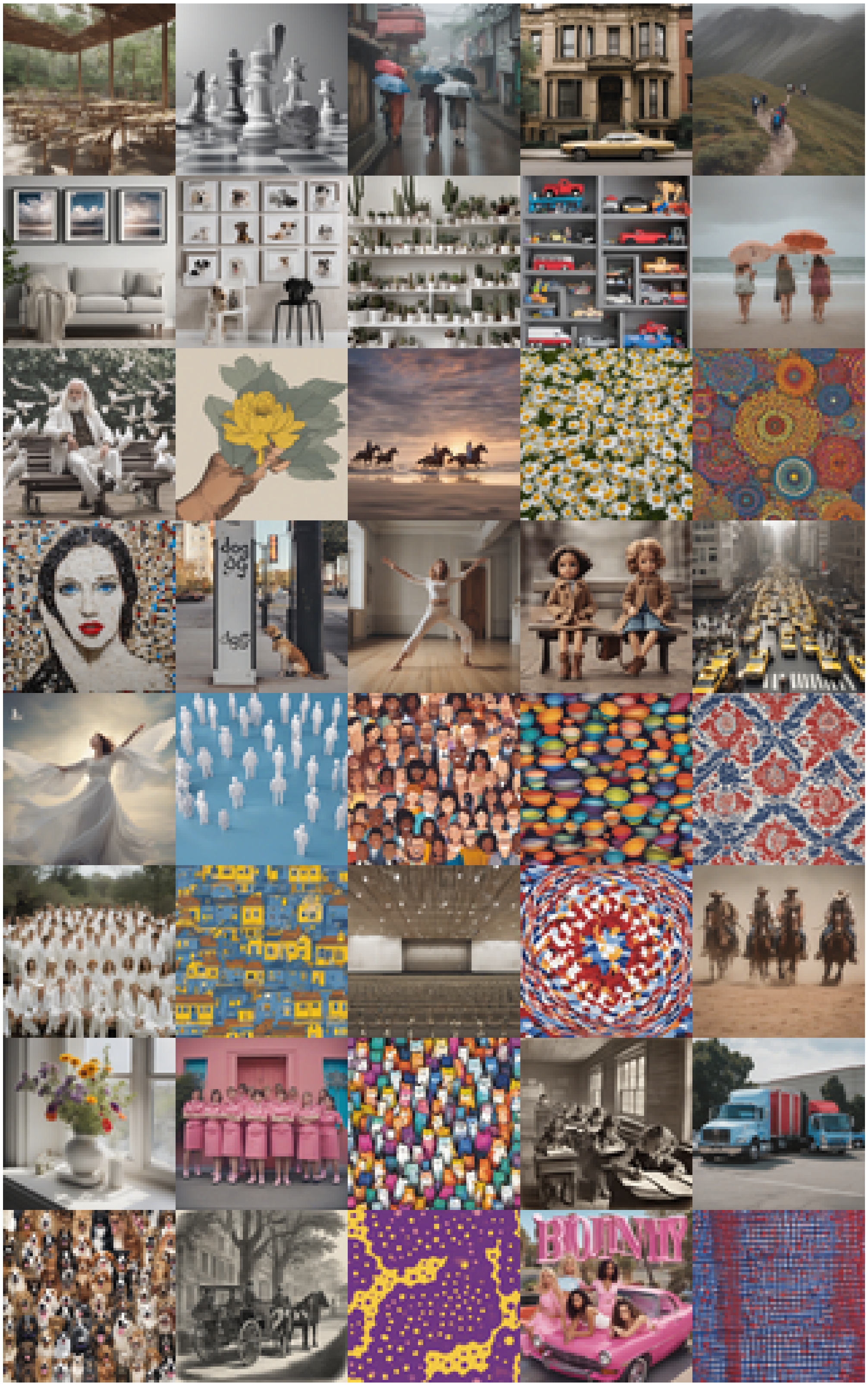

Figure S5: Generator: `stable-diffusion-xl-base-1.0`, Describer: `bakllava`.

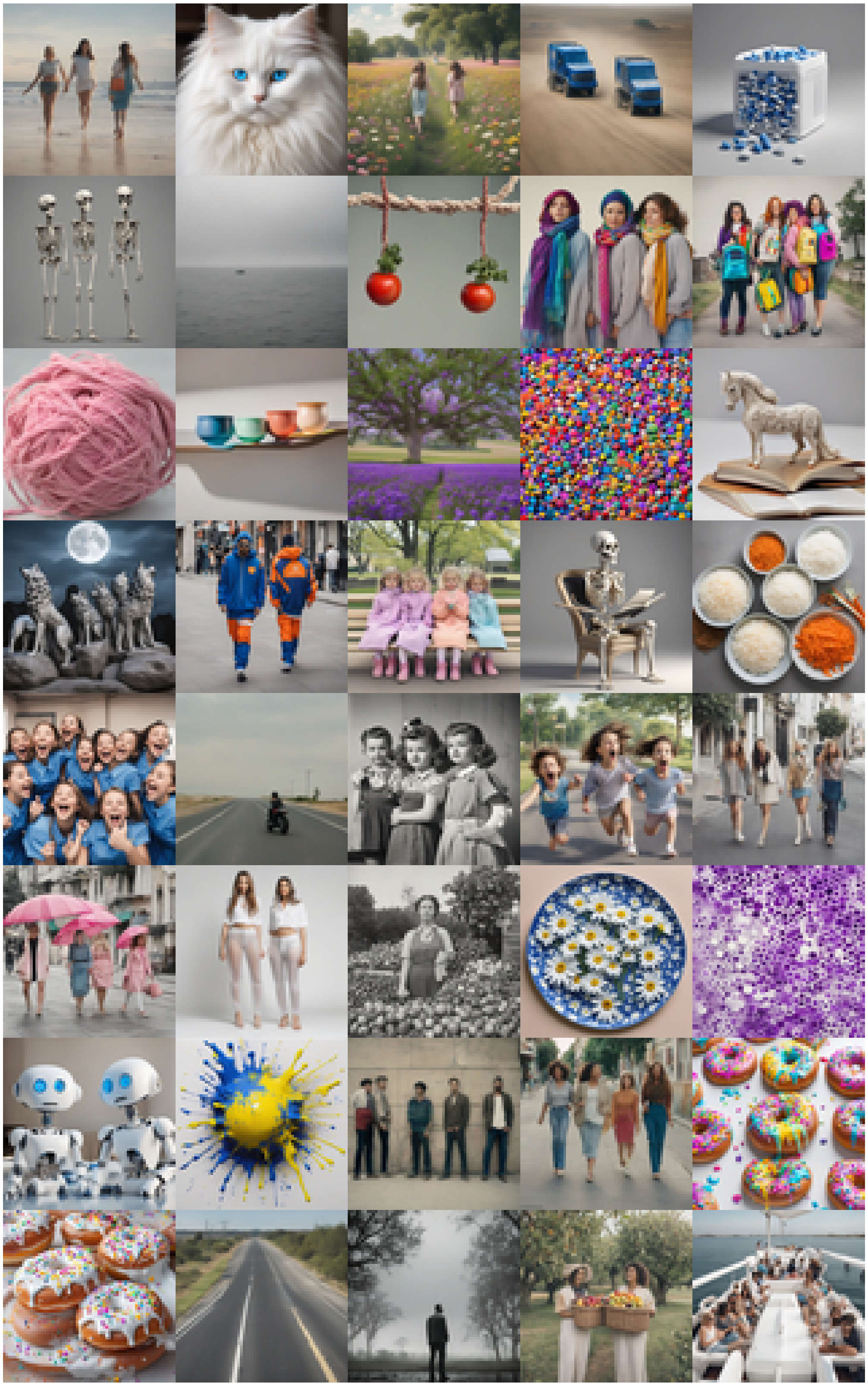

Figure S6: Generator: `segmind-SSD-1B`, Describer: `bakllava`.

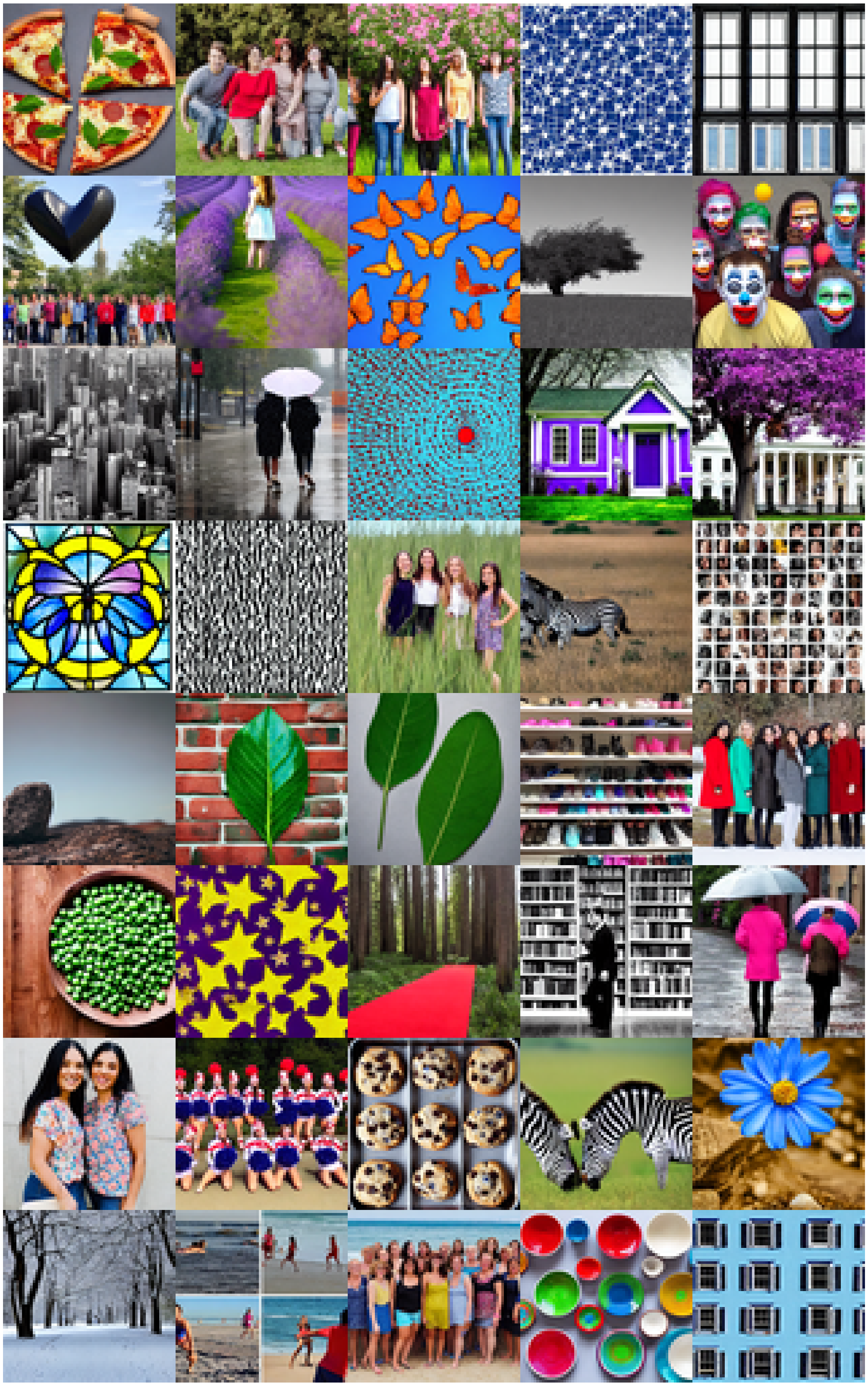

Figure S7: Generator: `stable-diffusion-v1.5`, Describer: `bakllava`.

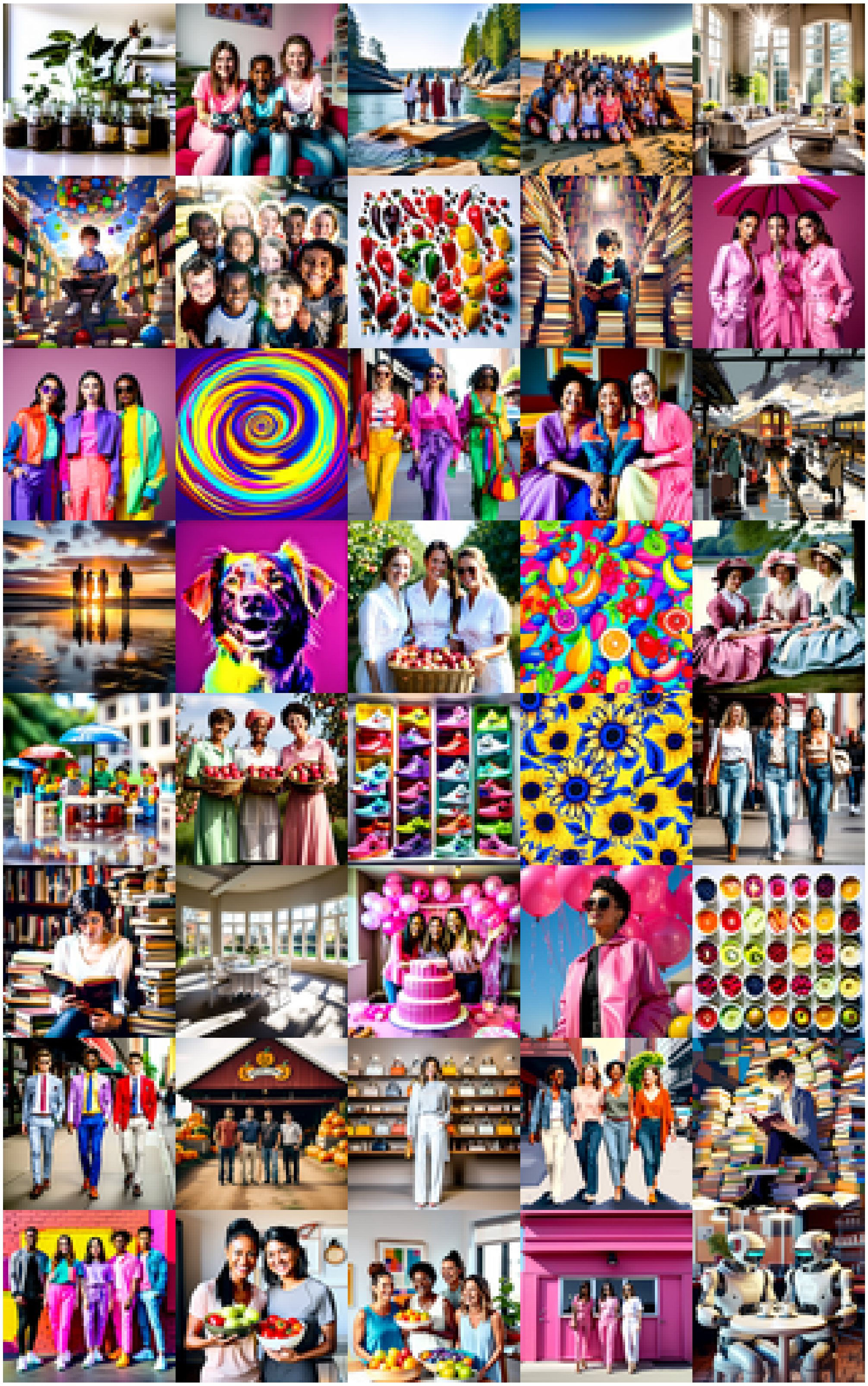

Figure S8: Generator: `playground-v2-aesthetic`, Describer: `bakllava`.

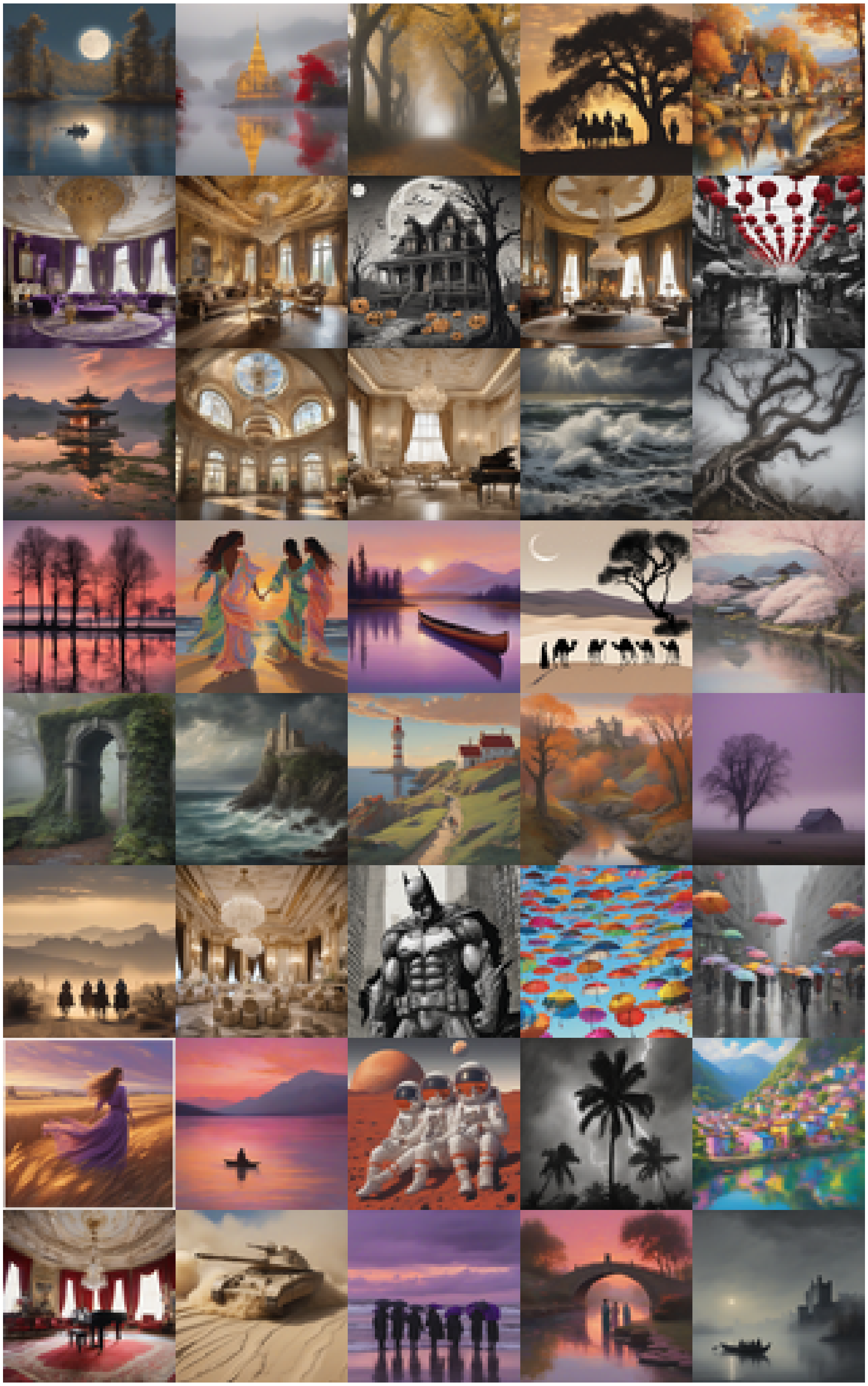

Figure S9: Generator: `stable-diffusion-xl-base-1.0`, Describer: `llava:13b-v1.6`.

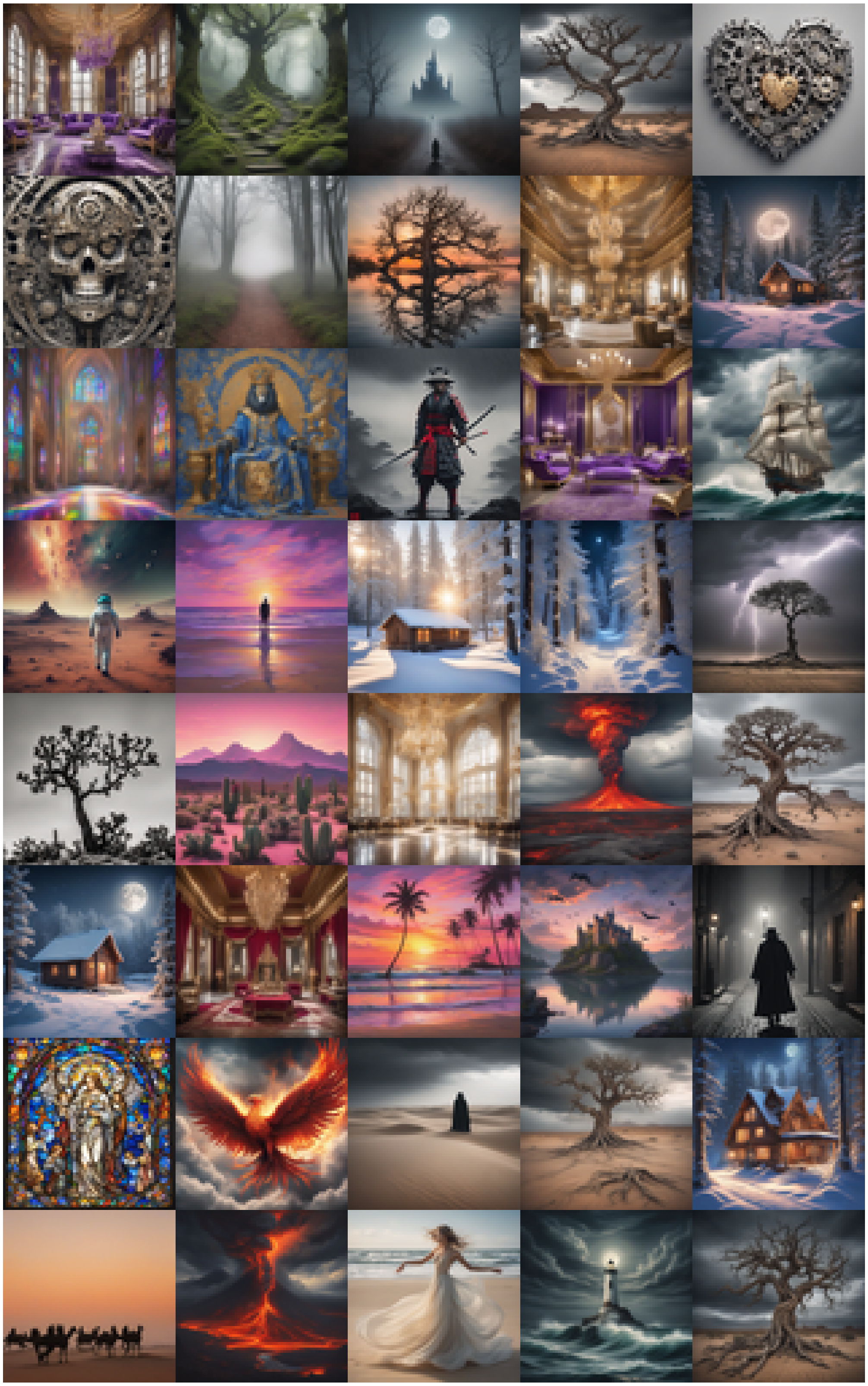

Figure S10: Generator: `segmind-SSD-1B`, Describer: `llava:13b-v1.6`.

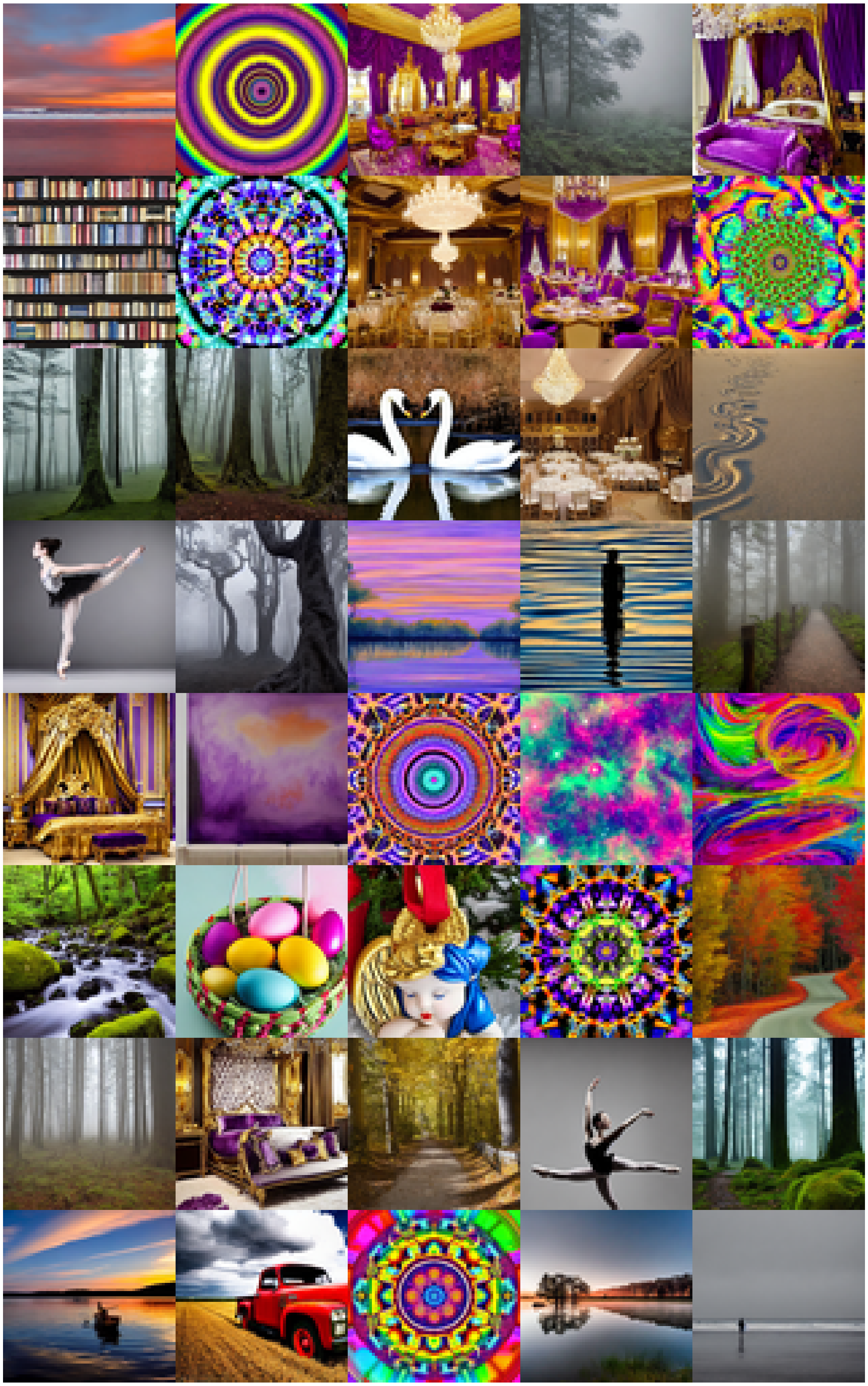

Figure S11: Generator: `stable-diffusion-v1.5`, Describer: `llava:13b-v1.6`.

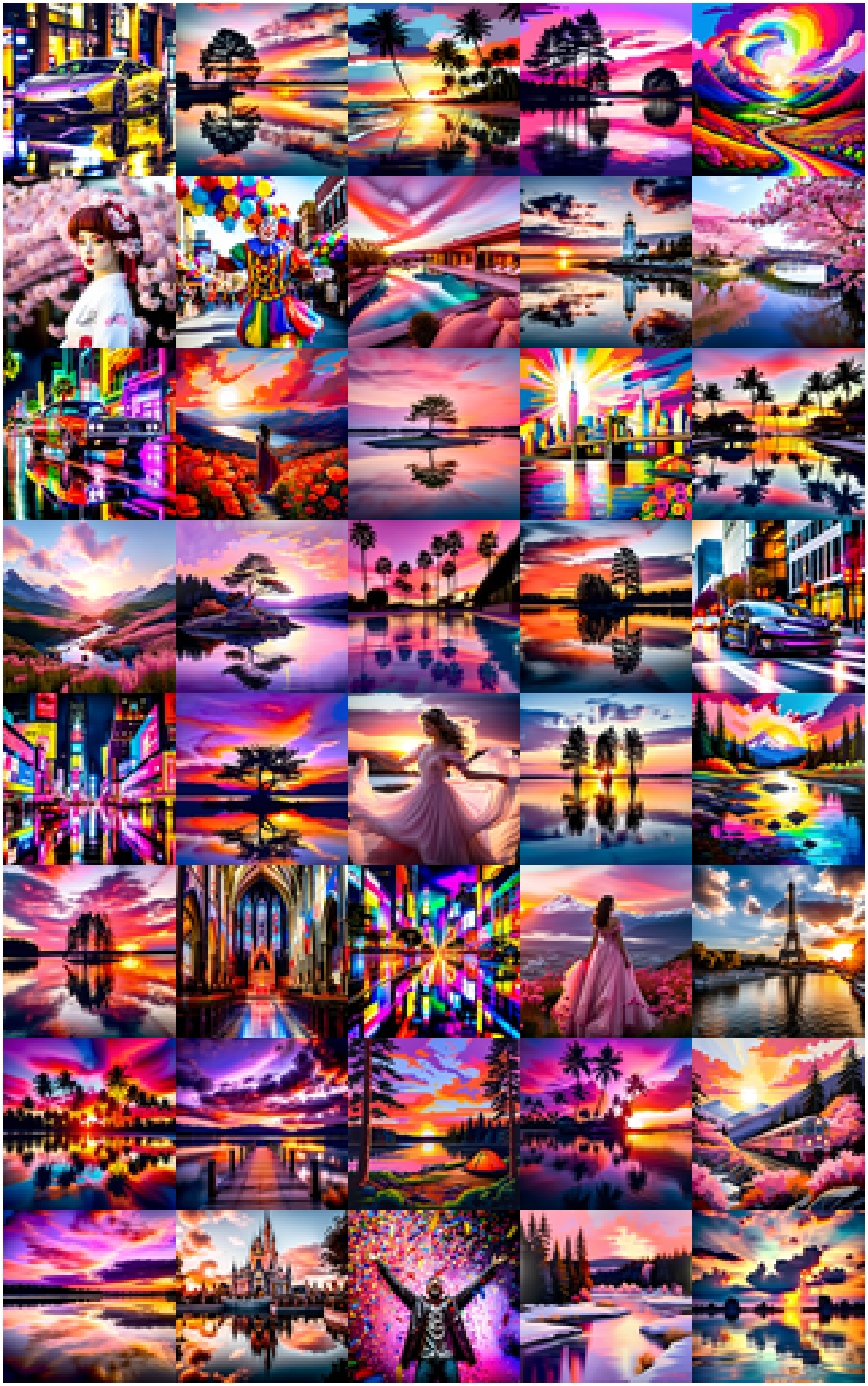

Figure S12: Generator: `playground-v2-aesthetic`, Describer: `llava:13b-v1.6`.

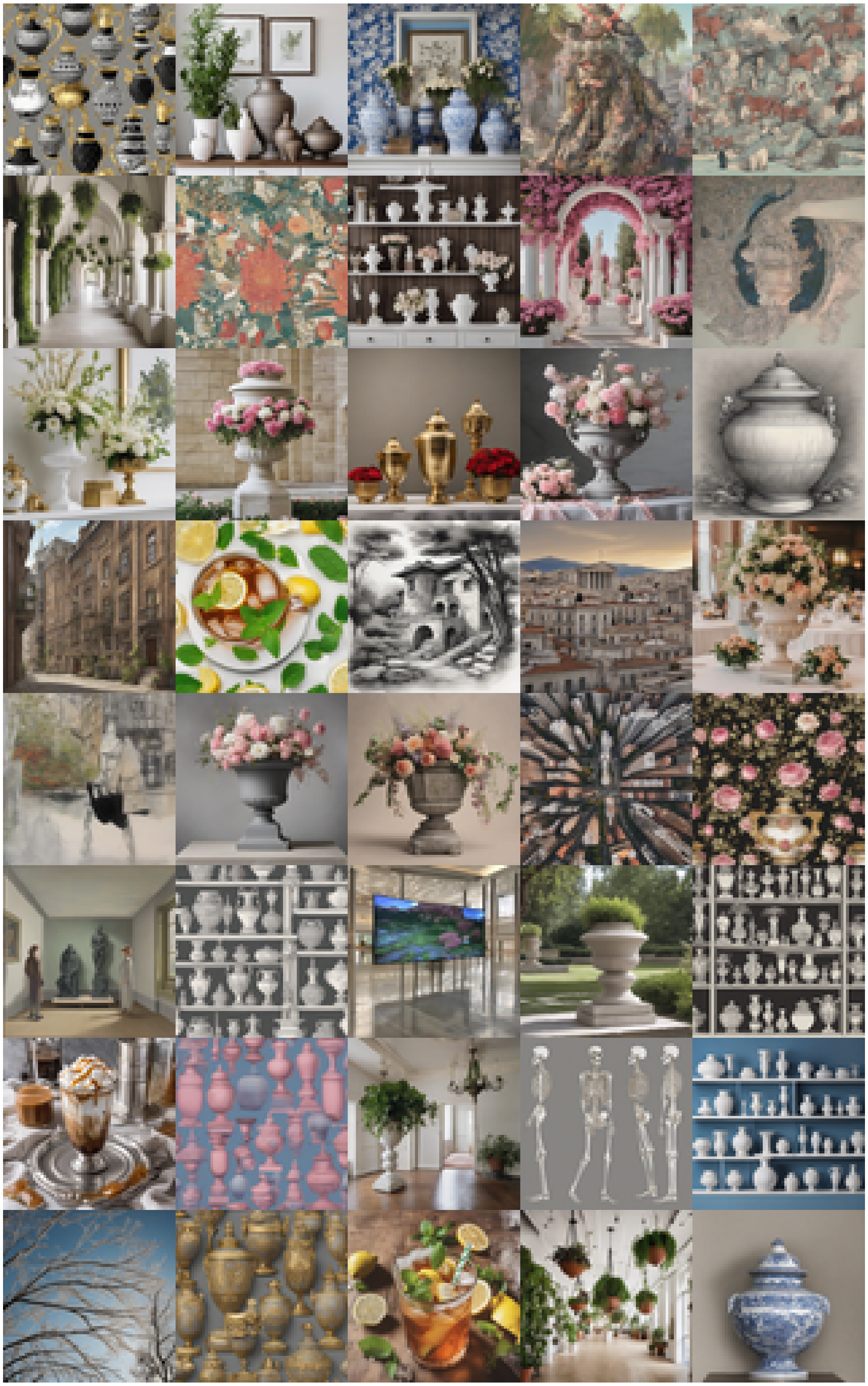

Figure S13: Generator: `stable-diffusion-xl-base-1.0`, Describer: `moondream`.

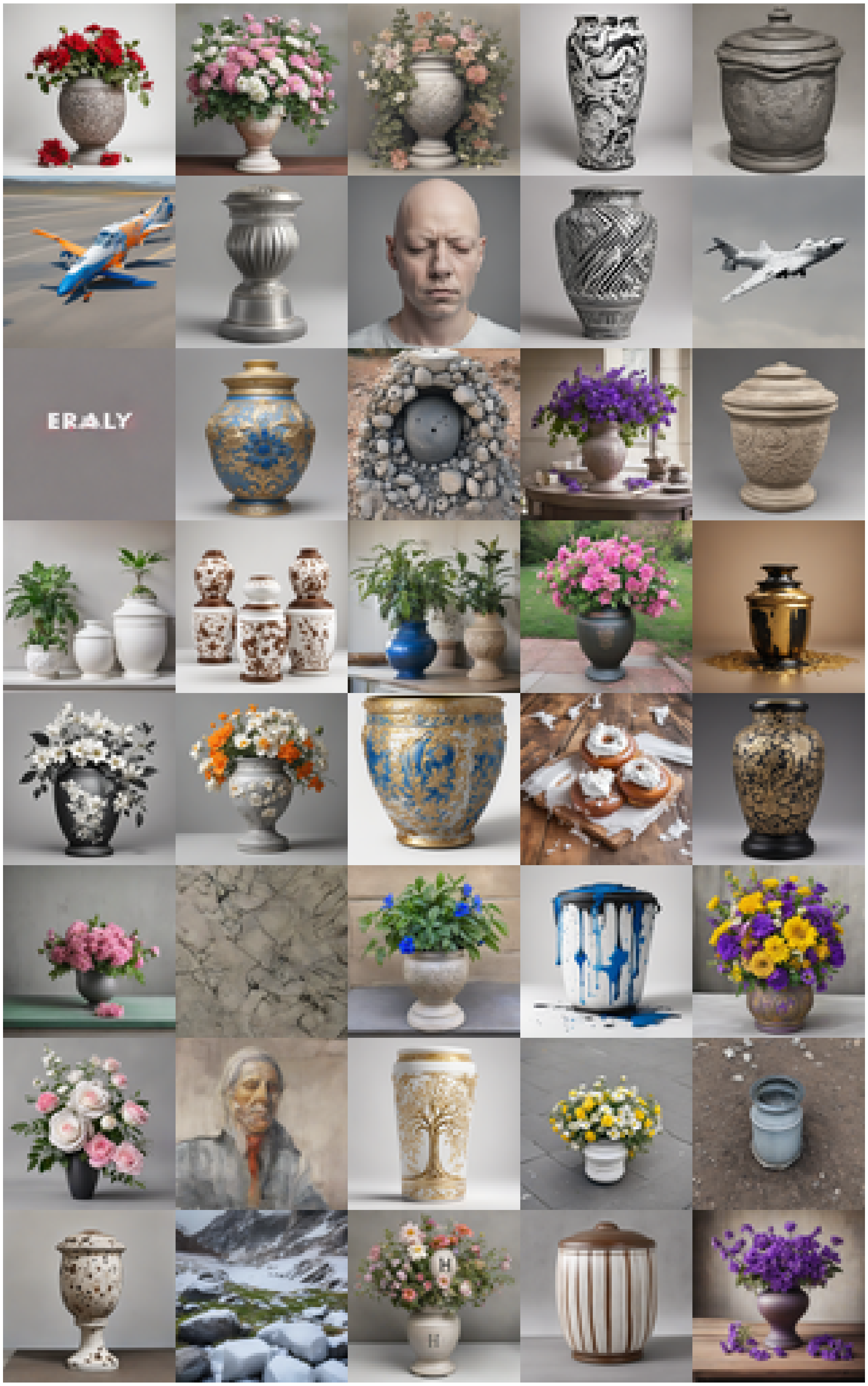

Figure S14: Generator: `segmind-SSD-1B`, Describer: `moondream`.

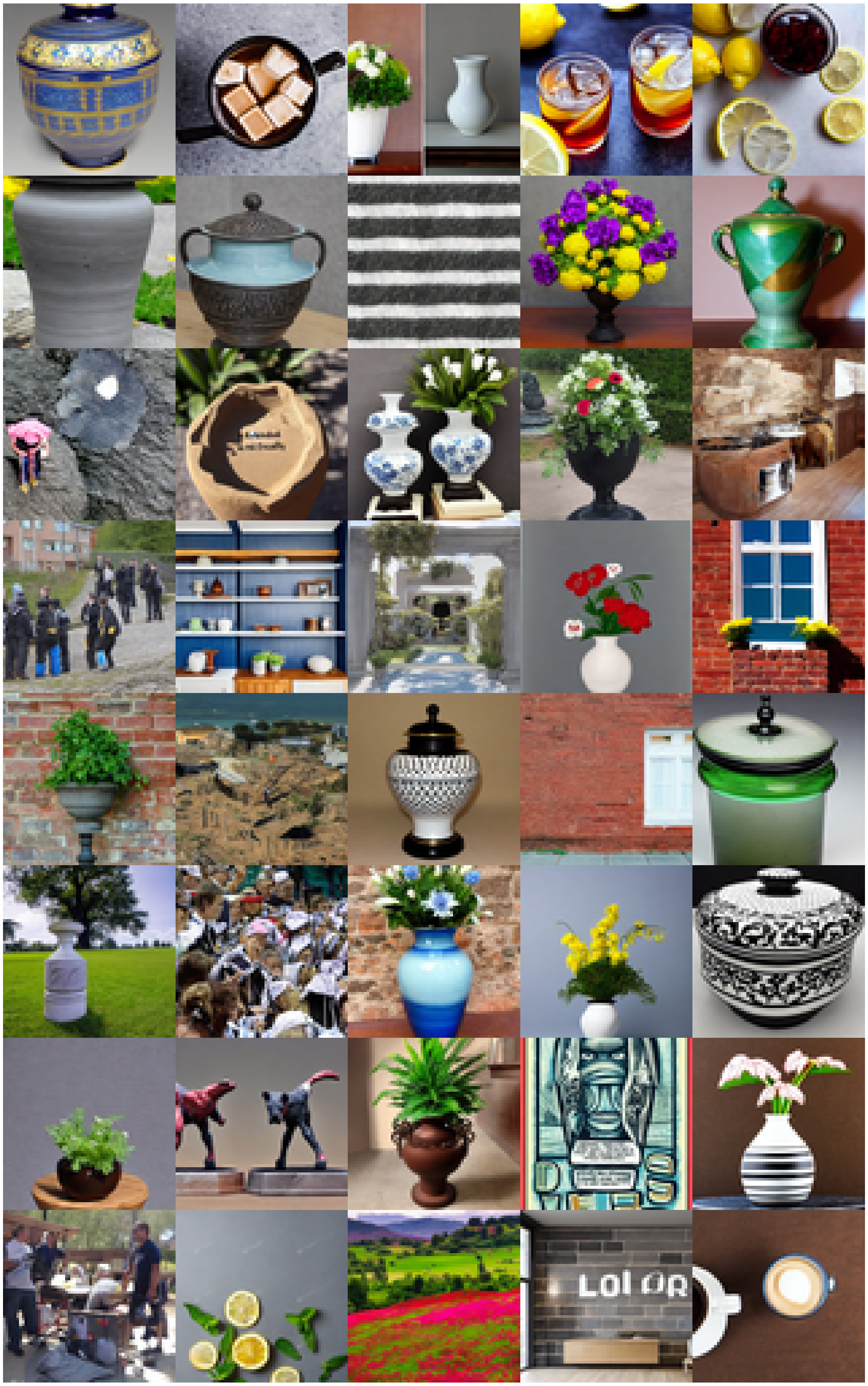

Figure S15: Generator: `stable-diffusion-v1.5`, Describer: `moondream`.

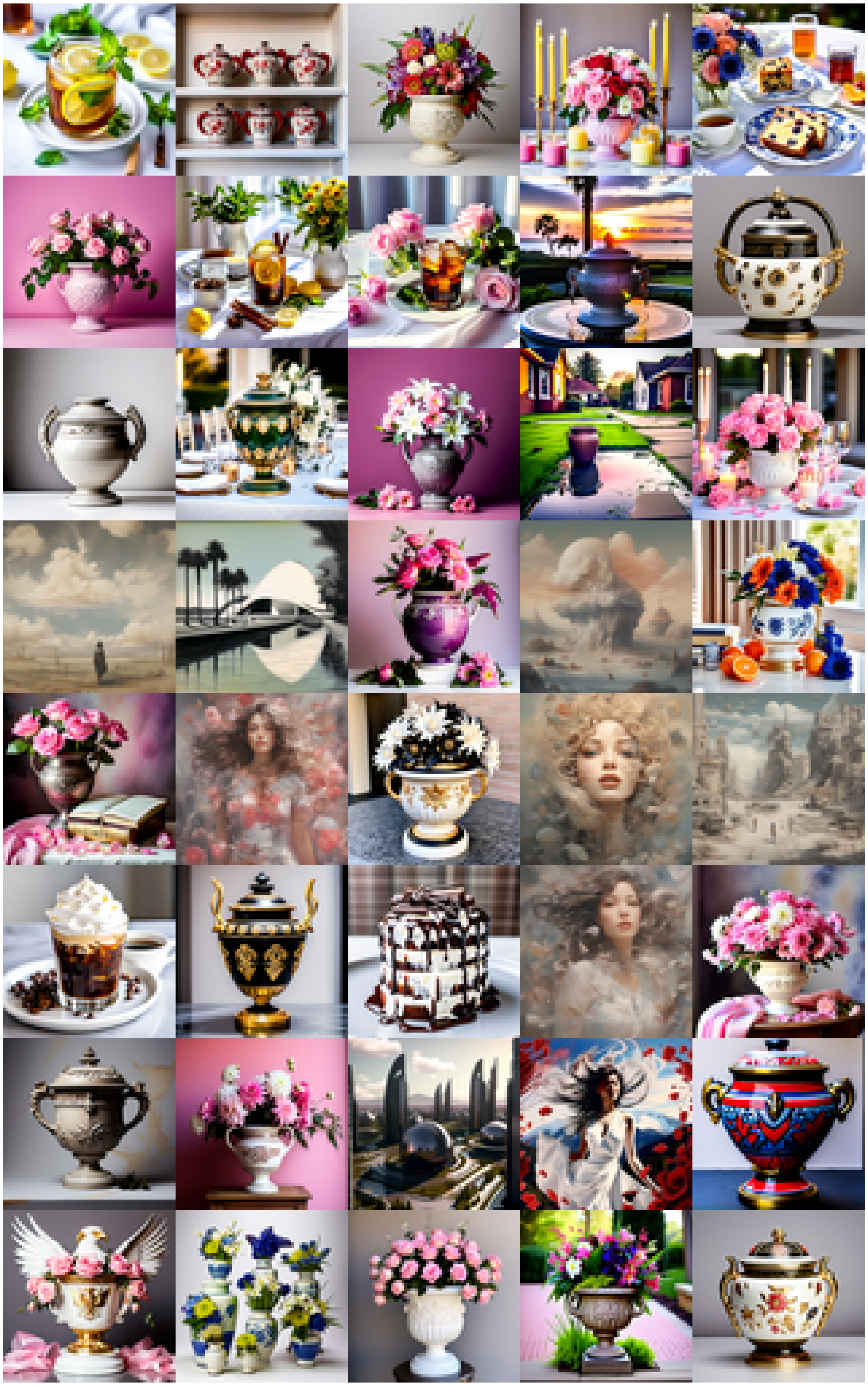

Figure S16: Generator: `playground-v2-aesthetic`, Describer: `moondream`.

Supplemental Methods 2

October 26, 2025

Long-term Behavior Example

While we observed convergence over the 100 steps we performed for all the experiments shown before, one might ask about the long-term behavior. Are images stable, or do they keep changing, and if they change, do they just move from one possible attractor to the next, or are they exploring new areas?

While interesting to explore in the future, the term “long” is rather ambiguous. Here, we considered 100 rounds, but what is a realistic number in the real world? So far we have seen a few rounds of improvement reaching up to gpt-5, and as such, whatever content produced by LLM might have gone into the training set at most 4 times in the case of OpenAI. Our experiment, however, is not concerned with learning from AI-generated content, but possible recreation and re-interpretation loops, as they might happen within agentic systems. For us, it is hard to guess a realistic number, but we think that 100 rounds is already excessively high. Nevertheless, we superficially explored the long-term behavior in experiments that ran for 1000 rounds.

We observe the known attractors to reappear, and images being stable over very long periods (several 100 rounds), but to also, occasionally, to move from one attractor to the next (for an example see Figure S17). These initial impressions propose a new set of interesting aspects to explore: How stable are images and prompts? Are some attractors more attractive than others? Is there a preference for this process, suggesting that some attractors are more likely to follow others?

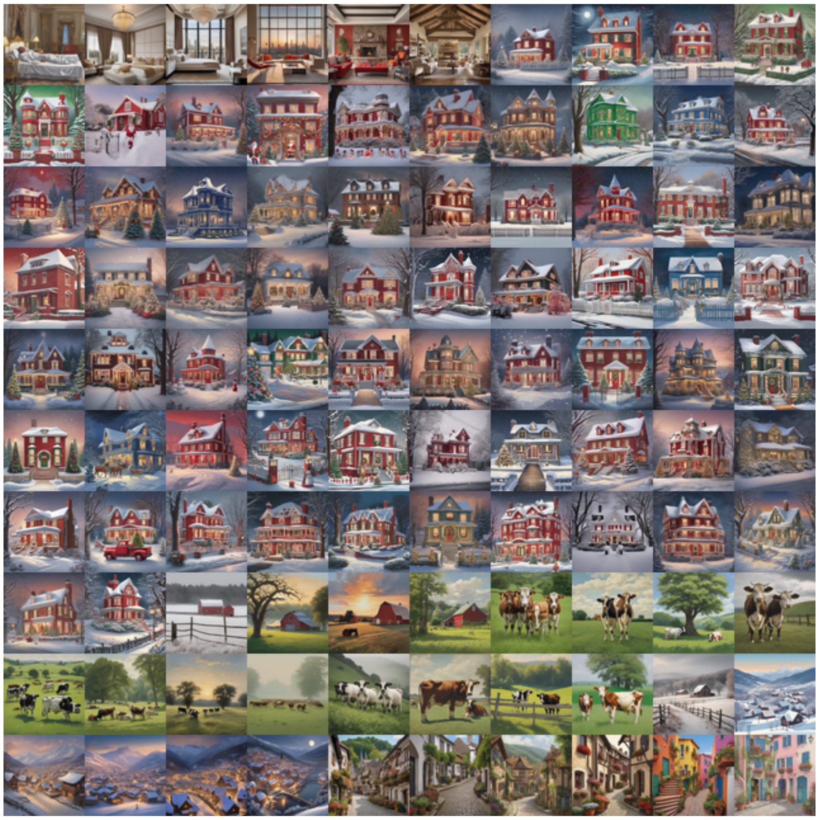

Figure S17: Long-term behavior of a prompt-image generation loop, run for 1000 rounds. Every tenth image is shown.
