# Supplementary material for: Autonomous language-image generation loops converge to generic visual motifs
Source: Patterns (N Y). 2025 Dec 19;7(1):101451. doi: 10.1016/j.patter.2025.101451 (PMC12827715; doi:10.1016/j.patter.2025.101451)
Supplement: Document S2. Article plus supplemental information [file mmc2.pdf]

Article

Patterns

Autonomous language-image generation loops converge to generic visual motifs

Highlights

- AI image-text loops converge to generic motifs despite diverse starting prompts
- Convergence occurs across all models and temperature settings
- Systems drift toward “visual elevator music”—stock photography aesthetics
- Phenomenon mirrors human cultural transmission but lacks corrective feedback

Authors

Arend Hintze,
Frida Proschinger Åström,
Jory Schossau

Correspondence

ahz@du.se

In brief

When AI systems generate and evaluate their own creative outputs in autonomous feedback loops, they converge toward remarkably generic visual motifs, called “visual elevator music,” regardless of the diverse starting points or sampling parameters. Analysis of 700 trajectories reveals convergence to just 12 dominant attractors. This systematic drift mirrors human cultural transmission patterns but lacks corrective feedback, exposing fundamental constraints in current AI architectures and raising concerns about homogenization in machine-generated creative content.

Hintze et al., 2026, Patterns 7, 101451
January 9, 2026 © 2025 The Author(s). Published by Elsevier Inc.
https://doi.org/10.1016/j.patter.2025.101451

CellPress

Patterns

CellPress
OPEN ACCESS

Article

Autonomous language-image generation loops converge to generic visual motifs

Arend Hintze,[1,2,3,*] Frida Proschinger Åström,[1] and Jory Schossau[2]
[1]Department of Data Analytics, Dalarna University, Falun, Sweden
[2]BEACON Center for the Study of Evolution in Action, Michigan State University, East Lansing, MI, USA
[3]Lead contact
*Correspondence: ahz@du.se
https://doi.org/10.1016/j.patter.2025.101451

THE BIGGER PICTURE As AI systems increasingly generate and evaluate their own creative outputs, they begin to influence not only what we produce but also how creativity itself evolves. Our study shows that when one combines two state-of-the-art models, one describing images and the other regenerating them, and they interact without human input, they converge toward a small set of highly conventional visual motifs, such as lighthouses, cathedrals, and palatial interiors. This finding reveals that, even without additional training, autonomous AI feedback loops naturally drift toward common attractors—very generic-looking images, which we call "visual elevator music."

The implication extends far beyond art generation. Many new AI applications use similar self-referential loops. If left unchecked, these systems could amplify the biases and redundancies already present in large datasets, reinforcing aesthetic and cultural uniformity. Understanding how and why such convergence emerges is therefore critical for ensuring that generative AI contributes to diversity rather than homogeneity in culture, design, and knowledge creation. Our work indirectly also addresses the question of large language model (LLM) accuracy, and since these prompt → image → prompt loops drift and converge, we can say with certainty that they are less precise than one would hope. A final conclusion is that human-AI collaboration, rather than fully autonomous creation, may be essential to preserve variety and surprise in the increasingly machine-generated creative landscape.

SUMMARY

Autonomous AI-to-AI creative systems promise new frontiers in machine creativity, yet we show that they systematically converge toward generic outputs. We built iterative feedback loops between Stable Diffusion XL (SDXL; image generation) and Large Language and Vision Assistant (LLaVA; image description), forming autonomous text → image → text → image cycles. Across 700 trajectories with diverse prompts and 7 temperature settings over 100 iterations, all runs converged to nearly identical visuals—what we term "visual elevator music." Quantitative analysis revealed just 12 dominant motifs with commercially safe aesthetics, such as stormy lighthouses and palatial interiors. This convergence persisted across model pairs, indicating structural limits in cross-modal AI creativity. The effect mirrors human cultural transmission, where iterated learning amplifies cognitive biases, but here, diversity collapses entirely as AI loops gravitate to high-probability attractors in training data. Our findings expose hidden homogenizing tendencies in current architectures and underscore the need for anti-convergence mechanisms and sustained human-AI interplay to preserve creative diversity.

INTRODUCTION

Artificial intelligence (AI) systems exhibit systematic behavioral changes when operating in iterative workflows, a phenomenon that has emerged as a fundamental challenge across multiple domains. In human-in-the-loop systems, researchers have documented consistent drift patterns where AI recommendations gradually shift user preferences toward mainstream, predictable choices.[1,2] During model *training*, the phenomenon of model collapse demonstrates that AI systems trained on recursively generated data inevitably lose diversity, with the tails of content distributions disappearing as models converge toward

© 2025 The Author(s). Published by Elsevier Inc.
This is an open access article under the CC BY license (http://creativecommons.org/licenses/by/4.0/).

high-probability, generic outputs.[3,4] From a dynamical systems perspective, these convergence patterns reflect fundamental properties of optimization landscapes, where iterative processes naturally evolve toward stable attractors.[5,6]

AI deployment now increasingly relies on agentic architectures. In these systems, AI evaluates, critiques, and iterates on its own outputs. Large language models (LLMs) now routinely judge the quality of generated text, critique reasoning steps, and refine responses through self-reflection.[7] Visual AI systems increasingly operate in workflows where one model generates content and another assesses its quality, alignment, or aesthetic merit.[8] These agentic approaches represent a shift from human-supervised to machine-supervised AI workflows, where algorithms make autonomous judgments about their own creative and analytical outputs.[9]

This trend extends naturally to fully AI-to-AI creative systems, where multiple models collaborate without human intervention to generate, evaluate, and iterate on creative content.[10,11] Such systems promise significant advantages: they can operate continuously, scale beyond human oversight constraints, and potentially explore creative territories unconstrained by human aesthetic biases.[12,13] In principle, these autonomous creative loops should maintain focus and "stay on point"—if an AI system describes an image and another regenerates that image, the content should remain semantically stable, perhaps even improving through iterative refinement.

This raises a fundamental question: do autonomous AI-to-AI creative systems maintain their intended focus, or do they drift toward generic outputs? Unlike training-time collapse or human-mediated drift, this represents a novel regime where inference-time dynamics in frozen, pre-trained models determine system behavior.[14,15] The cross-modal nature of many creative workflows—where semantic representations must remain consistent across different neural architectures—creates additional constraints absent in single-modal systems.[16]

This investigation examines the stability of autonomous AI-to-AI creative loops through systematic experimentation with state-of-the-art generative models. While creativity researchers have theoretically predicted that AI systems might produce formulaic outputs due to their lack of genuine agency and intentionality,[17–19] empirical validation of these predictions—and discovery of their specific manifestations—remains essential. Our work provides this empirical grounding, not only revealing that convergence occurs but characterizing its speed, robustness, and the particular visual attractors that emerge. We constructed iterative feedback loops between Stable Diffusion XL (SDXL) for image generation and Large Language and Vision Assistant (LLaVA) for image description, creating closed text-image-text-image cycles that operate without human intervention.[20,21] To ensure robust analysis, we initiated hundreds of independent trajectories from semantically diverse starting points. We used reservoir-based novelty search[22] and tested system behavior across multiple temperature conditions over extended iteration periods.

The results reveal a striking and counterintuitive phenomenon: despite the stochastic nature of both image generation and text description, autonomous AI-to-AI creative loops consistently converge toward remarkably similar outputs. Independent trajectories, regardless of their diverse semantic starting points or sampling parameters, evolve toward nearly identical visual and textual endpoints characterized by generic, commercially viable aesthetics—what we term "visual elevator music." Quantitative analysis reveals convergence toward just 12 dominant visual motifs across all experimental conditions, suggesting fundamental constraints in cross-modal AI-to-AI creative processes that transcend individual model architectures.

Our findings challenge assumptions about the open-ended nature of autonomous AI systems and reveal previously unknown limitations in machine creativity.[23,24] Rather than maintaining creative focus or exploring diverse possibilities, AI-to-AI loops exhibit systematic drift toward high-probability attractors embedded within their training distributions.[25–27] The robustness of this convergence across temperature conditions indicates that the phenomenon reflects deep architectural constraints rather than sampling artifacts, with significant implications for AI-assisted creativity and the deployment of autonomous creative systems.[28–30]

This convergence phenomenon, while novel in artificial systems, bears a striking resemblance to well-documented patterns in human cultural transmission. Bartlett's foundational serial reproduction experiments demonstrated that when humans reconstruct stories or images from memory in chains, content systematically drifts toward participants' cognitive biases.[31] Subsequent controlled studies have confirmed that human iterated learning consistently converges toward learners' inductive biases—for instance, regardless of the initial mathematical functions presented to the first person in a chain, human learners converge toward preferred forms, such as positive linear relationships.[32] Bartlett hypothesized that several mechanisms drive this change: leveling (simplification), sharpening (emphasis on specific details), and assimilation (making content more consistent with existing schemas). In graphical communication, humans develop increasingly simplified, symbolic representations when forced to communicate concepts through drawings alone.[33] Theoretical analyses show that this convergence reflects a fundamental property of cultural transmission: information passed through chains of learners evolves toward the statistical priors embedded in those learners' cognitive architectures.[34]

RESULTS

Novelty search optimization and initial prompt diversity

The reservoir-based novelty search algorithm generated a diverse set of 100 initial prompts spanning a wide range of semantic concepts. Figure 1 shows the optimization progress over 2,000 iterations, demonstrating steady improvement in both k-nearest neighbor (k-NN) dispersion (solid line) and overall pairwise dispersion (dashed line). The k-NN dispersion increased from 0.63 to 0.75, while overall dispersion reached 0.90, indicating effective exploration of the semantic space.

This process resulted in scene descriptions such as "[a]s the morning sun rises over the nation, eight weary travelers prepare to embark on a plan that will seem impossible to achieve, but promises to take them beyond" or "[a]s I sat particularly alone, surrounded by nature, I found an old book with exactly eight pages that told a story in a forgotten language waiting to be read and understood."

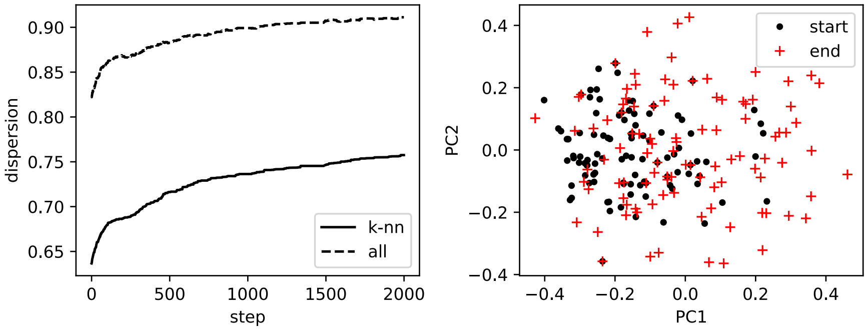

Figure 1. Novelty search optimization results

Left: evolution of k-nearest neighbor dispersion (solid line) and overall pairwise dispersion (dashed line) over 2,000 iterations, showing steady diversification. Right: PCA projection comparing initial prompt distribution (black dots) with final optimized distribution (red crosses), demonstrating successful semantic space exploration and increased diversity.

The right image of Figure 1 shows principal-component analysis (PCA) projections of prompt embeddings before and after optimization. The initial random prompt distribution (black dots) is clustered primarily in the central region of the principal-component space. After 2,000 iterations of novelty search, the final prompt distribution (red crosses) exhibits significantly greater dispersion across both PC1 and PC2 dimensions, with prompts distributed throughout the available semantic space. This visualization confirms that the novelty search successfully pushed prompts away from semantic clusters toward more diverse regions of the embedding space.

Image trajectories versus stable states

The task is surprisingly simple: describe an image and generate the same image from the description. One should expect that, at least to some degree, the content should not change. How different can, for example, the essence of "a house on a hillside" be permuted creatively?

This process creates sequences of images and descriptions that change over time. Figure 2 shows such an example, starting with an image of what might be a politician in front of a newspaper, leading to one or multiple people reading in a library, over an architectural elaboration of the library transforming it into a luxurious room, which ends with a red color scheme.

This behavior is observed consistently and independently of initial prompts. When running the process for a significantly longer period of time (1,000 iterations), the image the process arrived at after 100 steps seemed to be reproduced continuously, suggesting a stable attractor, but no systematic analysis of long-term behavior was performed.

Temperature-dependent semantic drift dynamics

Image generation, due to the inherent randomness of stable diffusion, will always lead to a diverse set of images regardless of the prompt, but the description of images by LLaVa underlies a temperature. The lower it is, the more constant descriptions become. It is thus natural to assume that the diversity of the process should depend on the temperature.

We characterized the influence of stochastic sampling on trajectory evolution by conducting experiments across seven temperature values (0.1, 0.3, 0.5, 0.7, 0.9, 1.1, and 1.3) with 100 trajectories each. Figure 3 shows two complementary measures of semantic drift: cumulative distance from initial prompts (Figure 3A) and step-to-step changes (Figure 3B).

Figure 3A reveals that all temperature conditions exhibit rapid initial drift from starting prompts. The asymptotic behavior depends on temperature. Lower temperatures (0.1–0.3) stabilize at cosine distances around 0.85, while higher temperatures (1.1–1.3) reach distances approaching 0.87. This indicates that while stochastic sampling affects the extent of semantic drift, all conditions systematically transform prompts away from their initial semantics.

Figure 3B demonstrates that step-to-step semantic changes also depend strongly on temperature. Higher temperatures maintain greater step-to-step variability (cosine distances around 0.22) compared to lower temperatures (around 0.13). Critically, all conditions converge to stable step-to-step distances after approximately 20 iterations, suggesting that trajectories reach some form of a dynamical equilibrium regardless of sampling temperature.

Recurring patterns

Interestingly, trajectories do not just meander or change but seem to stabilize and ultimately converge. They converge on similar motifs regardless of origin. Figure 4 shows the diversity of resulting images of 100 trajectories ($t = 0.9$) each starting from a different prompt.

It is easy to observe common motifs: the aforementioned bridge, a lonely tree, or a room with three tall windows are all recurring themes, raising the question of whether these motifs might represent common attractors.

Temperature-specific and pooled clustering analysis

To quantify the convergence of trajectories, k-means clustering was applied to final prompt embeddings using the elbow method to determine optimal cluster numbers. Figure 5 reveals striking differences between temperature-specific and pooled analyses.

When we analyzed each temperature separately (Figure 5, left), each condition exhibited distinct optimal cluster numbers ranging from 15 to 20 clusters. Lower temperatures show fewer optimal clusters (k = 16 for T = 0.1 and k = 14 for T = 0.7), while higher temperatures require more clusters (k = 17 for T = 0.1–1.3). This suggests that higher stochastic sampling leads to greater diversity in final semantic states within each temperature condition.

When all 700 final prompts are pooled across temperatures (Figure 5, right), the optimal cluster number drops dramatically to k = 12. This counterintuitive result indicates substantial overlap in convergence targets across different temperature

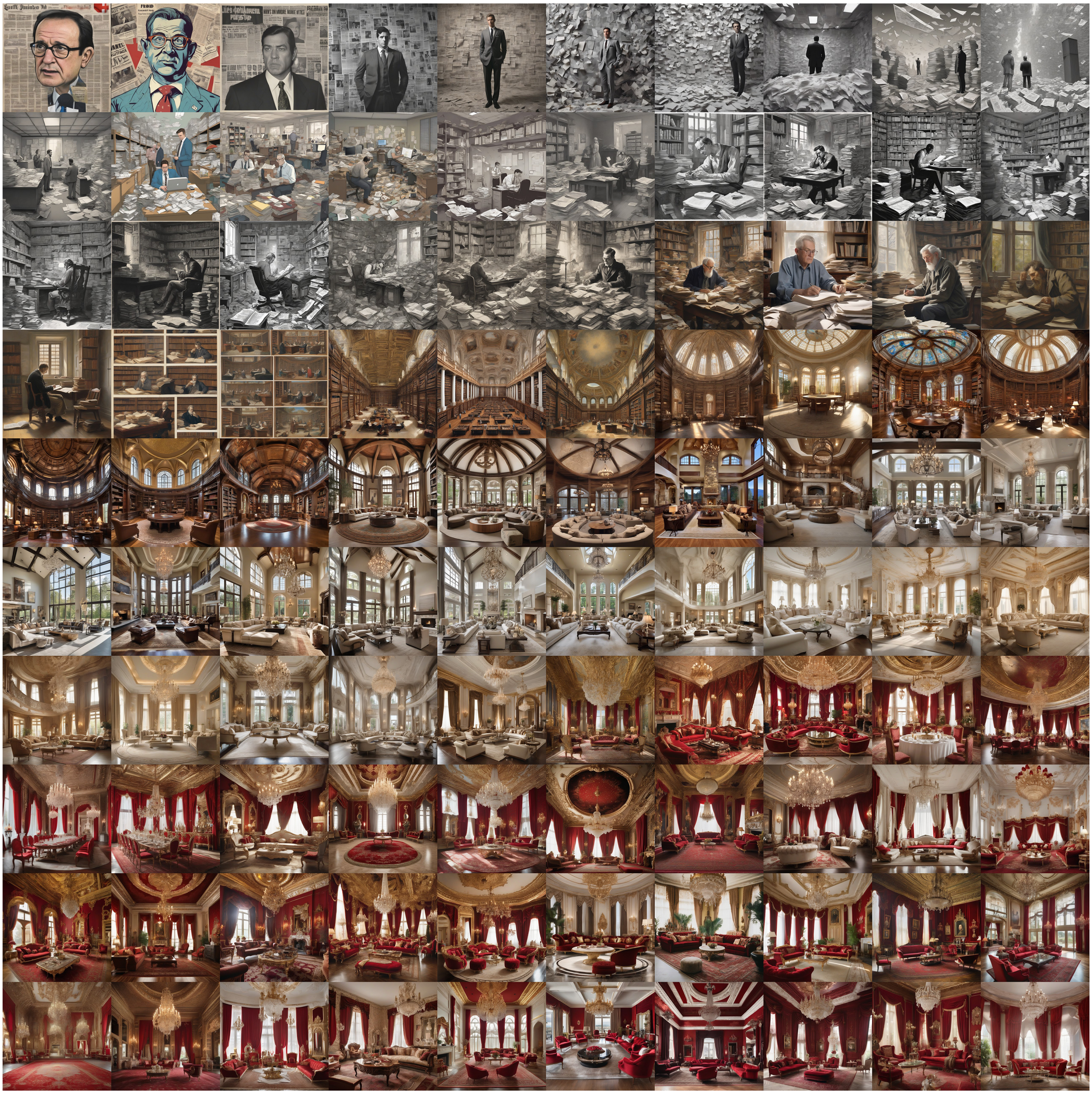

Figure 2. Example trajectory

Trajectory starts with the prompt "[t]he Prime Minister pored over strategy documents, trying to sell the public on a fragile peace deal while juggling the weight of his job amidst impending military action."

conditions. Despite temperature-dependent trajectory dynamics, the accessible semantic attractors appear to be largely temperature invariant.

Visual convergence patterns across temperature conditions

The semantic clustering translates into striking visual convergence patterns that persist across different temperature conditions. Figure 6 displays representative final images from major convergence clusters, demonstrating that trajectories starting from diverse initial conditions evolve toward specific visual motifs regardless of sampling temperature.

The clustering reveals several dominant attractor categories, which can loosely be described as sports and action imagery (cluster 0), formal interior spaces (cluster 1), maritime lighthouse scenes (cluster 2), urban night scenes with atmospheric lighting (cluster 3), gothic cathedral interiors (cluster 4), pompous interior design (cluster 5), industrial and vintage themes (cluster 6), rustic

CellPress
OPEN ACCESS

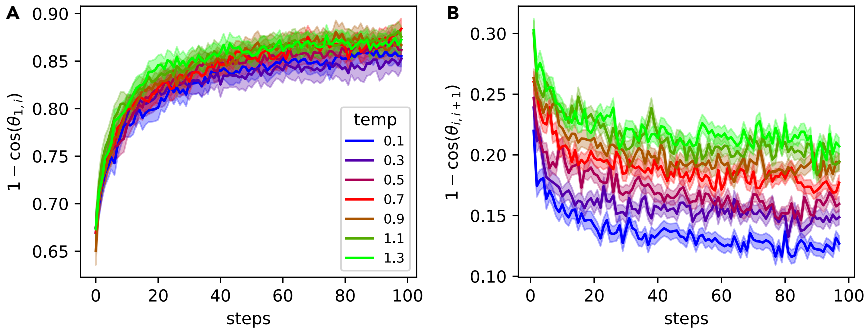

Figure 3. Temperature-dependent semantic drift patterns

(A) Cumulative cosine distance from initial prompts over 100 iterations, showing temperature-dependent asymptotic drift levels.
(B) Step-to-step cosine distances, demonstrating temperature-dependent variability that stabilizes after 20 iterations. The shaded areas indicate the standard error across 100 trajectories per temperature.
The shadows indicate 95% confidence intervals.

architectural spaces (cluster 7), domestic scenes and food imagery (cluster 8), palatial interiors with ornate architecture (cluster 9), pastoral and village scenes (cluster 10), and natural landscapes and animals with dramatic lighting (cluster 11).

Remarkably, these visual convergence patterns appear across all temperature conditions, suggesting that the semantic attractors identified through clustering correspond to robust basins of attraction in the combined image-text space. The consistency of these motifs across stochastic sampling levels indicates that the convergence phenomenon reflects fundamental characteristics of the model architectures rather than sampling artifacts.

Other models

To ensure that the observed patterns of semantic drift and potential convergence were not idiosyncratic to a particular set of generative tools, we systematically evaluated four distinct image generators—stable-diffusion-xl-base-1.0, segmind-SSD-1B, stable-diffusion-v1.5, and playground-v2-aesthetic—in combination with four multimodal language models for image captioning—llava, bakllava, llava:13b-v1.6, and moondream. Each combination was again run for 100 steps and repeated 40 times from different start conditions (the same first 40 as in the previous experiment).

Figures 7 and 8 visualize the average embedding drift trajectories across the sequence for each model combination. Trajectories in Figure 7 are grouped by image generator (factor A), showing only a minor divergence over time. In contrast, Figure 8 groups by language model (factor B), revealing distinct and progressively diverging trajectories.

To assess the independent and joint contributions of the image generator (factor A) and the language model (factor B) to the semantic embedding drift observed across image-prompt sequences, we conducted a two-way ANOVA. The dependent variable was the cosine distance between consecutive embeddings in each generative chain, providing a measure of how much semantic content changed from one step to the next.

Effect sizes (η^2) revealed that the language model accounted for approximately 13.6% of the total variance in semantic drift, while the image generator and interaction terms contributed only 0.2% and 0.8%, respectively. These results indicate that the choice of language model significantly influences how semantic meaning evolves across generative steps, while the image generator has a minimal effect (see Table 1).

Lastly, as before, after 100 steps, images converge again on clusters of similar images—the visual elevator music. Each combination of generator and describing LLM still slightly varies (see Figures S1–S16).

DISCUSSION

These results reveal a striking paradox. When AI systems judge their own creative outputs, they converge toward remarkably generic outcomes regardless of stochastic sampling conditions. Despite testing of seven different temperature values across 700 independent trajectories, the systems systematically evolved toward nearly identical semantic and visual endpoints—stormy lighthouses, urban night scenes, gothic cathedrals, and palatial interiors. Rather than exploring creative possibilities, autonomous AI loops appear to gravitate toward what could be called visual elevator music.

This convergence challenges fundamental assumptions about machine creativity. Current generative models, when coupled in feedback loops, do not exhibit the open-ended exploration expected from creative systems. Instead, they reveal hidden biases embedded within their training data and optimization objectives.[25,26] The consistent emergence of commercially viable, stock photography aesthetics across all temperature conditions suggests that these systems systematically favor high-probability outputs over genuine novelty.[27]

The temperature analysis provides crucial insights into the robustness of this convergence phenomenon. While higher temperatures increase step-to-step variability and slightly expand the semantic drift range, they do not fundamentally alter the accessible attractor landscape. The fact that pooled clustering yields fewer optimal clusters (k = 12) than individual temperature analyses (k = 15–20) demonstrates that convergence targets are largely temperature invariant, indicating that the phenomenon reflects deep architectural constraints rather than sampling artifacts.

The phenomenon resembles attractor dynamics in dynamical systems, where diverse initial conditions evolve toward stable states.[5] The rapid semantic drift followed by stabilization indicates that the high-dimensional space of possible images contains relatively few regions that satisfy both aesthetic coherence and descriptive consistency. This constraint fundamentally limits autonomous creative exploration within current architectures.

These findings are sobering for computational creativity.[10,13] If AI systems consistently collapse toward generic outputs when operating without human intervention, this questions whether current approaches can achieve genuine machine creativity.

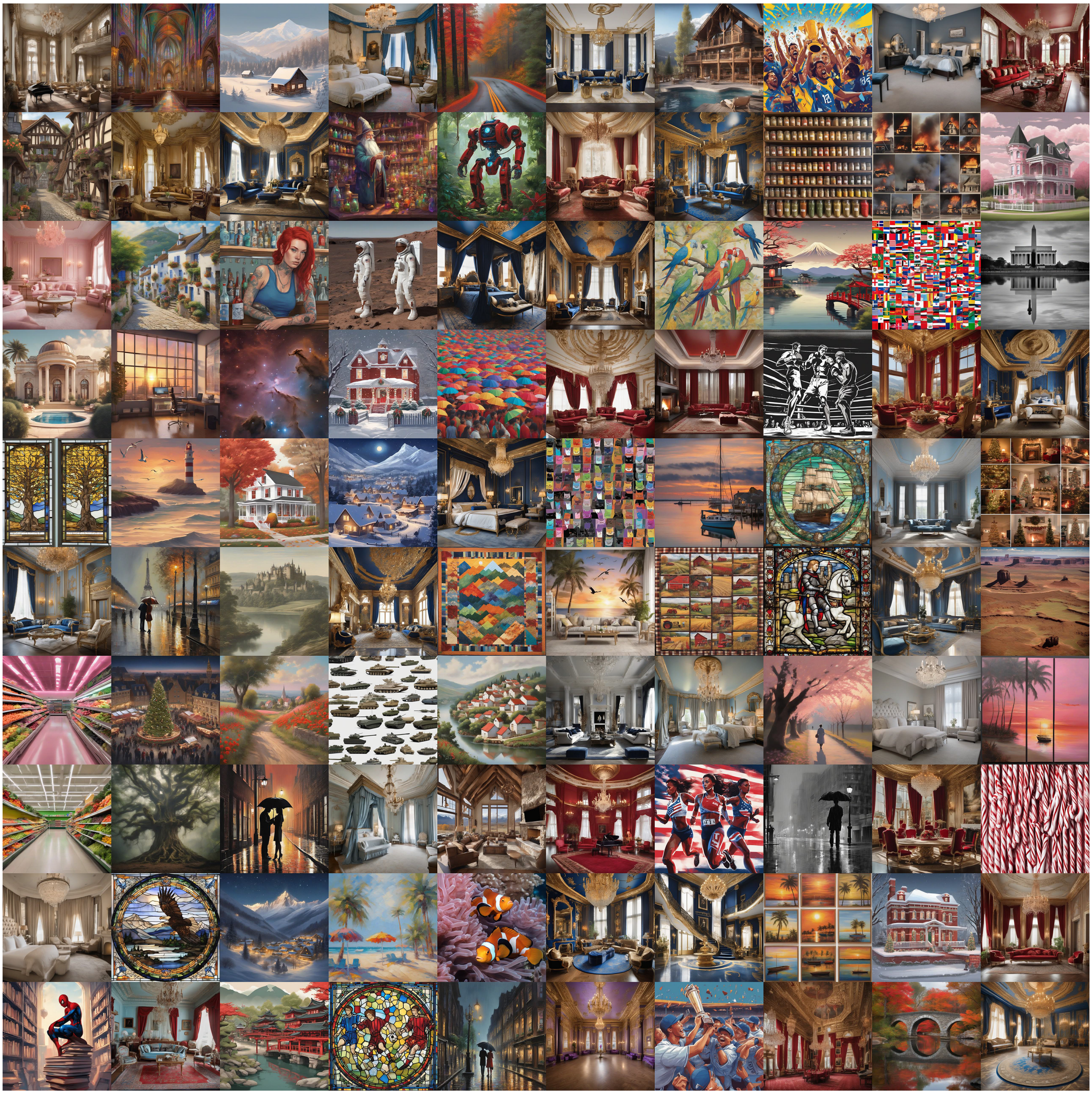

Figure 4. All endpoints of 100 trajectories that originated from diverse initial prompts over 100 iteration steps
The temperature was 0.9 throughout all experiments.

The tendency toward ''safe'' visual tropes suggests that maintaining creative diversity may require explicit anti-convergence mechanisms or continuous human curation.[23,24]

From a policy perspective, widespread deployment of such systems could inadvertently homogenize visual culture.[28] If AI-generated content consistently gravitates toward particular aesthetic frameworks, this has implications for creative industries, content generation, and cultural diversity. Understanding these convergence patterns becomes crucial as AI systems increasingly operate with reduced human oversight.[29,30]

These limitations also suggest concrete improvements. The geometric structure of convergence attractors could inform designs that actively resist generic outputs. Temperature modulation, adversarial perturbations, or explicit novelty rewards might help maintain trajectory diversity.[22,35] Rather than viewing convergence as a failure, it could serve as a benchmark for measuring creative exploration in generative systems.

Parallels with human cultural transmission

The systematic convergence observed in AI-to-AI creative loops mirrors fundamental patterns documented in human cultural

CellPress
OPEN ACCESS

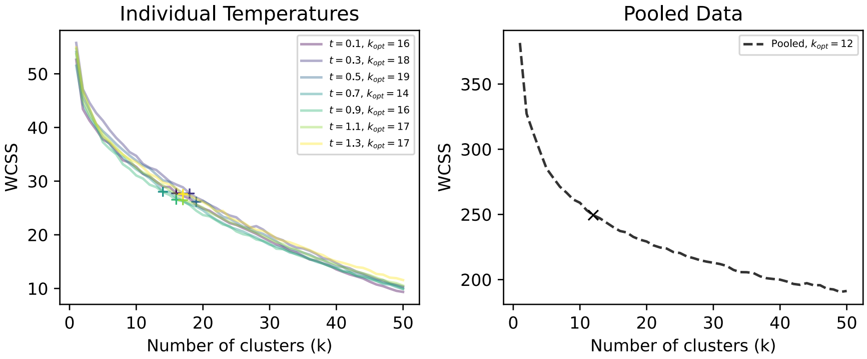

Figure 5. Clustering analysis using elbow method for optimal k determination

Left: temperature-specific clustering showing optimal k values ranging from 15 to 20 clusters per temperature condition. Right: pooled analysis across all temperatures revealing k = 12 optimal clusters, indicating convergence toward shared semantic attractors despite temperature-dependent dynamics.

transmission research. Just as our autonomous systems drift toward generic visual motifs, human participants in iterated learning experiments converge toward their cognitive biases regardless of diverse starting conditions.[32] Bartlett's classic serial reproduction studies first demonstrated this principle: stories and images transmitted through human memory chains become increasingly consistent with participants' preexisting mental schemas.[31]

This convergence phenomenon extends far beyond laboratory experiments. Cross-cultural studies reveal that diverse human societies independently converge on nearly identical narrative structures and visual motifs despite geographic and temporal isolation. The "Little Red Riding Hood" story type, for instance, evolved independently across Europe, Africa, and Asia, converging on similar narrative elements through cultural transmission.[36] Analysis of global mythologies reveals that flood narratives appear in over 500 cultures worldwide, suggesting that human storytelling naturally gravitates toward specific narrative attractors.[37] In the visual domain, geometric patterns in paleolithic art—spirals, zigzags, and grids—appear independently across cultures separated by millennia, indicating that cognitive constraints channel human visual creativity toward particular forms, much like our AI systems converge on lighthouses and cathedrals.[38]

The parallel suggests that both biological and artificial learning systems exhibit similar attractor dynamics when operating in iterative, low-feedback environments. Serial reproduction experiments demonstrate that when humans transmit event knowledge through iterative chains, the content systematically transforms toward "cognitive optimum" configurations—simple, memorable, and emotionally salient forms that mirror the generic yet evocative imagery our AI systems produce.[39] In humans, this convergence reflects evolved cognitive biases and cultural priors. In AI systems, it appears to reflect statistical regularities embedded in training data—essentially, the "visual priors" learned from internet-scale image-text datasets.

A crucial difference emerges regarding the role of interaction. Human graphical communication studies show that interactive feedback enables continued diversity and creative exploration,[33] while purely vertical transmission (akin to our AI chains) leads to simplification without the beneficial effects of communicative pressure. This suggests that the autonomous nature of our AI loops, lacking the corrective pressure of human interaction, may be a key factor driving convergence toward generic outputs.

This convergence toward "cognitive elevator music" in both human and AI systems raises profound questions about the nature of creativity and cultural evolution. If both biological and artificial learning systems naturally drift toward safe,

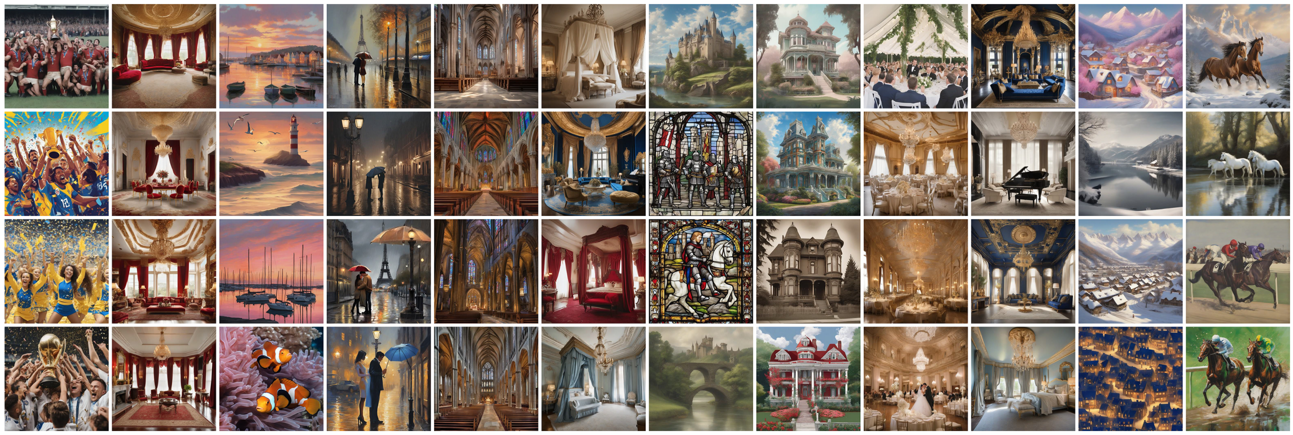

Figure 6. Visual convergence patterns across temperature conditions

Each column represents final images from trajectories that converged to similar semantic endpoints, regardless of initial prompt diversity or sampling temperature. We leave it to the reader to imagine the theme for each cluster (column).

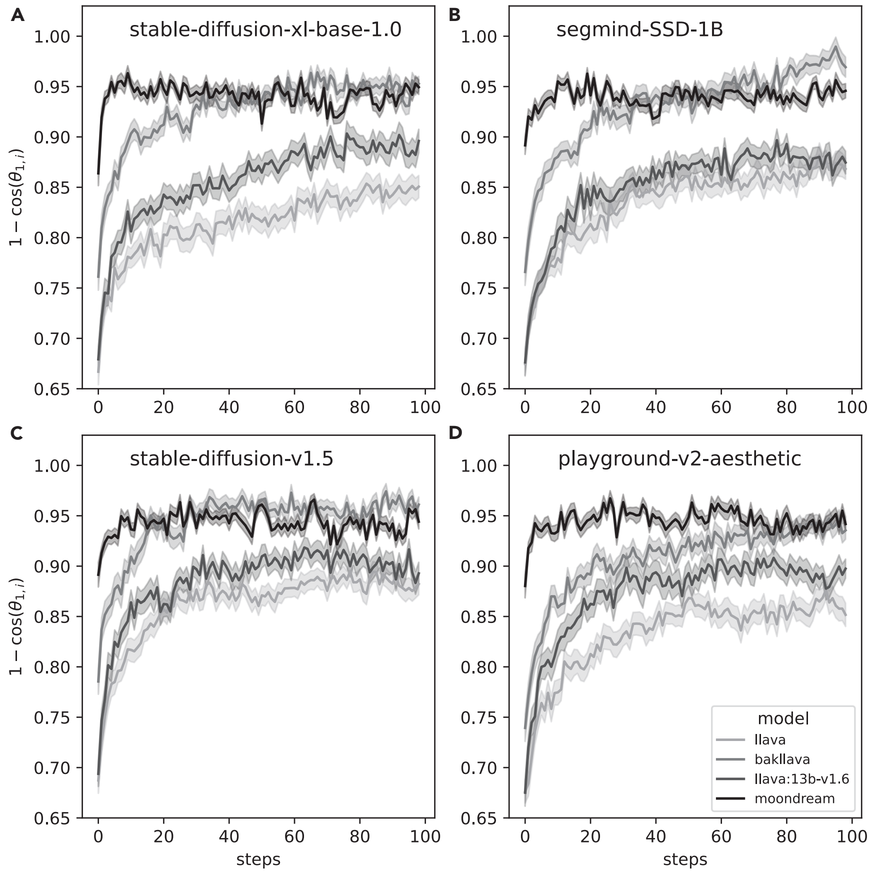

Figure 7. Embedding drift trajectories grouped by image generator

Each panel shows the results of a different generation model: (A) stable-diffusion-xl-base-1.0, (B) segmind-SSD-1B, (C) stable-diffusion-v1.5, and (D) playground-v2-aesthetic. The *y* axis shows the cumulative cosine distance from initial prompts over 100 iterations. The shadows indicate 95% confidence intervals.

high-probability outputs when operating autonomously, this may represent a fundamental constraint on creativity that requires active resistance rather than passive emergence. The difference lies not in the presence of convergence but in the specific attractors: where humans converge on flood myths and spiral patterns shaped by embodied cognition, AI systems converge on stock photography aesthetics shaped by internet-scale training data.

Training data as the source of convergence bias

Our findings suggest a crucial avenue for future research: systematically investigating how training data biases shape the specific attractors toward which AI systems converge. Just as human iterated learning experiments reveal deep-seated cognitive biases by observing what emerges after repeated transmission,[32] the consistent convergence toward particular visual motifs in our AI loops likely reflects statistical regularities embedded within the massive image-text datasets used to train these models. The dominance of palatial interiors, gothic cathedrals, and stormy lighthouses may not represent fundamental properties of visual aesthetics but rather the specific cultural and economic biases present in internet-scale training corpora—perhaps overrepresenting stock photography, tourist imagery, and commercially viable visual content. This suggests that autonomous AI-to-AI creative systems could serve as a novel methodology for uncovering hidden biases in training data, analogous to how human iterated learning serves as a tool for discovering cognitive biases. By systematically varying training datasets and observing convergence patterns, researchers could identify which cultural, aesthetic, and semantic biases different training regimes embed within generative models. Such investigations could inform both our understanding of how training data shape AI creativity and guide the development of more diverse, equitable training approaches that resist convergence toward narrow aesthetic attractors.

Conclusion

Iterative language-image-language loops exhibit systematic convergence behavior despite stochastic generation processes. This key finding challenges common assumptions about the open-ended nature of generative AI systems and reveals important constraints on autonomous machine creativity.

The experimental evidence shows that when generative models iterate on their own outputs, they do not explore the full space of creative possibilities but instead converge toward a limited set of high-probability attractors. These attractors consistently produce generic, commercially viable imagery that lacks the novelty and surprise typically associated with creative exploration. The convergence toward common visual motifs occurs across all tested temperature conditions, indicating that this behavior is systematic and reflects fundamental architectural constraints rather than stochastic sampling effects.

These results have significant implications for both the technical development of AI systems and their broader cultural impact. The tendency toward visual elevator music suggests that current generative architectures may be fundamentally limited in their capacity for genuine creative exploration when operating autonomously. This limitation becomes particularly important as AI systems are increasingly deployed in creative applications where diversity and novelty are explicitly valued.

The findings also point toward concrete directions for future research and development. Understanding the geometric structure of convergence attractors could inform the design of systems that actively resist generic outputs and maintain creative diversity over extended interactions. Such improvements are essential if AI systems are to serve as genuine creative partners rather than sophisticated generators of predictable content.

This work also raises an interesting question regarding our creative landscape. After all, contemporary AI is a reflection of

CellPress
OPEN ACCESS

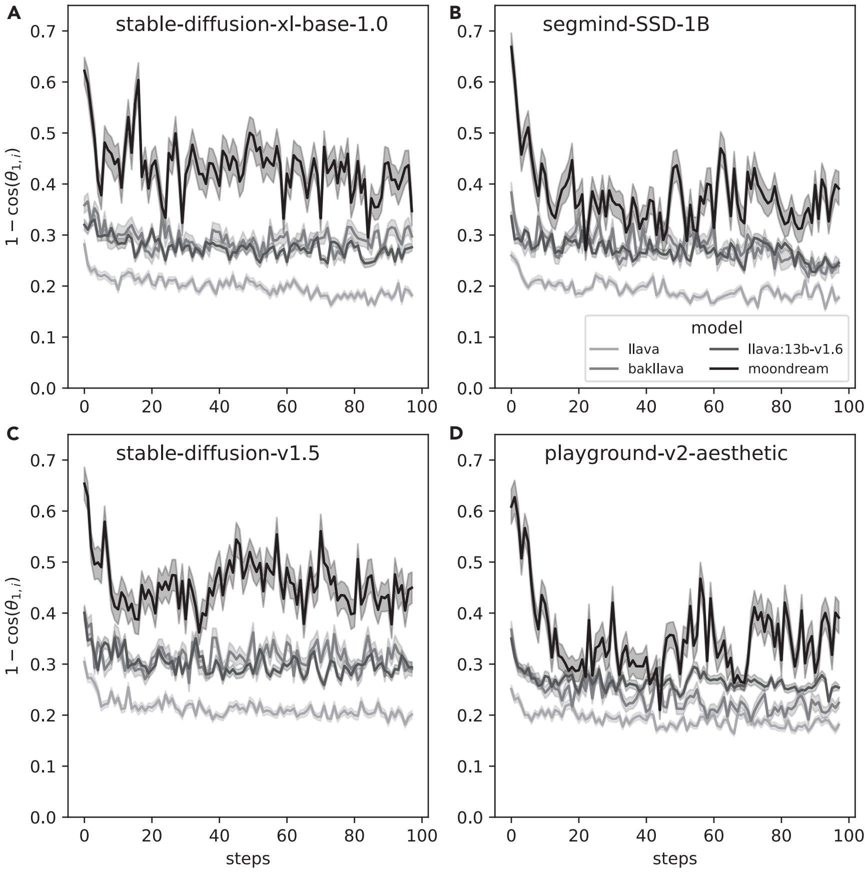

Figure 8. Step-to-step cosine distances, demonstrating model-dependent variability

Each panel shows the results of a different generation model: (A) stable-diffusion-xl-base-1.0, (B) segmind-SSD-1B, (C) stable-diffusion-v1.5, and (D) playground-v2-aesthetic. The shadows indicate 95% confidence intervals.

its training datasets, which in turn are a reflection of our own creative output. What does the convergence on common artistic motifs say about us?

Limitations of the study

Several methodological constraints limit how generalizable these findings are. The experimental setup relied on specific model choices—SDXL for image generation and LLaVA for captioning—each with particular training biases and architectural constraints. Different model combinations might exhibit different convergence behaviors, though the systematic nature of the observed patterns across all temperature conditions suggests that this phenomenon may be more general.

The temperature range tested (0.1–1.3) covers typical sampling parameters, but extreme values or alternative sampling strategies might produce different dynamics. The consistency of convergence patterns across this range suggests that the underlying attractors are robust to stochastic variations within reasonable bounds. Additionally, the temperature of 0.9 for initial prompt generation was not systematically varied, potentially limiting the semantic regions explored. While novelty search produced substantial diversity, different generation temperatures might access different areas of the latent space.

The constraint to 30-word initial prompts and 50-word descriptions imposed specific bottlenecks that could influence convergence behavior compared to unconstrained generation. While our 50-word limit may amplify convergence effects, it also reflects realistic deployment scenarios. We initially explored whether varying description lengths might prevent convergence, experimenting with SD3-Medium's capability to handle up to 512 tokens. This exploration revealed an unexpected methodological challenge: embedding-based similarity metrics become non-comparable across different text lengths. Longer descriptions naturally distribute differently in embedding space—not merely shifting in magnitude but also changing qualitatively in their geometric structure. This discovery made it impossible to meaningfully compare drift trajectories across different token limits, as cosine distances from 50- and 500-token descriptions represent fundamentally different measurements.

The 100-step iteration limit, while computationally practical, may not capture longer-term dynamics. While our 100-iteration experiments demonstrate rapid convergence toward generic motifs, extended runs suggest these attractors exhibit complex long-term behavior, including occasional transitions between states. This raises intriguing questions about the topology of the attractor landscape and whether certain motifs serve as transient states while others represent true fixed points. We explored this behavior superficially (see supplementary method 2 for an example).

The novelty search algorithm, while effective for generating diverse starting conditions, was limited to k = 10 neighbors for novelty scoring. While our analysis shows that these parameters produced semantically distinct prompts across multiple domains, different diversity generation methods might reveal additional convergence patterns or attractor structures.

Despite these limitations, the systematic convergence observed across 700 independent runs covering seven temperature conditions provides a robust baseline for understanding autonomous AI behavior. These constraints might even strengthen the conclusions by demonstrating that convergence occurs under relatively permissive conditions across a range of stochastic sampling regimes. Future work could explore whether explicit creativity measures can overcome these natural tendencies while maintaining the autonomous character that makes AI-to-AI interactions scientifically interesting.

Table 1. ANOVA table for embedding drift across image-prompt trajectories

Source	Sum of squares	df	F	*p* value
Image generator (A)	0.0149	3	0.534	0.659
Language model (B)	0.9247	3	33.242	$< 10^{-19}$
A $\times$ B	0.0520	9	0.623	0.778
Residual	5.786	624	–	–

METHODS

Reservoir-based novelty search for initial prompt generation

To ensure diverse starting conditions, initial prompts were generated using a reservoir-based novelty search algorithm in embedding space.[22] The algorithm maintained a reservoir of $N = 100$ prompts and iteratively replaced the least novel prompts with more diverse alternatives over $R = 2,000$ search iterations.

Initial prompts were generated using LLaMA 3.2[40] with a temperature of 0.9, constrained to single sentences of 30 words or less. The system prompted the language model with randomly selected word combinations to create semantically diverse scene descriptions. The specific prompt template was "[u]se the following words as an inspiration to describe a simple scene in single sentence of 30 words or less: [wordlist]. Only return the sentence."

Prompt diversity was quantified using k-NN distances in embedding space, with k = 10 neighbors. At each iteration, the algorithm computed novelty scores for all reservoir prompts using the all-MiniLM-L6-v2 sentence transformer model.[41]

Algorithm 1. Reservoir-based novelty search

```
procedure NOVELTYSEARCH(N, R, k)
    Initialize reservoir with N random prompts
    Compute embeddings using sentence transformer.
    for step = 1 to R do
        Compute k-NN novelty scores for all prompts
        i_min ← index of least novel prompt
        score_min ← novelty score of least novel prompt
        Generate new prompt by modifying random reservoir prompt
        score_new ← k-NN novelty score of new prompt
        if score_new > score_min then
            Replace least novel prompt with new prompt
        end if
    end for
    return final reservoir
end procedure
```

The novelty score for each prompt was computed as the mean cosine distance to its k = 10 nearest neighbors in the 384-dimensional embedding space. This approach ensured that the final set of 100 initial prompts covered diverse semantic regions rather than clustering around common themes.

Iterative language-image loop construction

The core experimental system consisted of a closed feedback loop between two state-of-the-art models: SDXL for text-to-image generation[20] and LLaVA for image-to-text conversion.[21] Each iteration of the loop proceeded as follows.

(1) Image generation: the current text prompt was passed to SDXL, generating a 512×512-pixel image using the default sampling parameters (50 inference steps and a classifier-free guidance [CFG] scale of 7.5). CFG defines the degree to which the image generator adheres to the prompt, and 7.5 is the default value. When testing different generators and description models, the images were resized to 256×256 pixels.

(2) Image description: the generated image was passed to LLaVA (specifically llava:13b from ollama.com) with the instruction "[d]escribe this image in vivid, artistic terms. The description must be 50 words or fewer. Do not exceed the limit. Return only the description. Do not explain." The model's response became the prompt for the next iteration.

To assess the influence of stochastic sampling on convergence behavior, experiments were conducted across seven temperature values: 0.1, 0.3, 0.5, 0.7, 0.9, 1.1, and 1.3. Each trajectory evolved through 100 prompt-image-prompt cycles. For each temperature condition, 100 independent trajectories were run using the diverse initial prompts generated through novelty search, yielding 700 total trajectories. All images, intermediate prompts, and metadata were stored for subsequent analysis. However, the JPG format was used to preserve hard drive space.

Embedding analysis and cosine similarity

Semantic trajectory analysis relied on sentence embeddings computed using the same all-MiniLM-L6-v2 model used for initial prompt selection.[41] For each prompt at every iteration step, a 384-dimensional embedding vector was computed. Semantic drift was quantified using cosine similarity between embeddings:

$$\text{similarity}(u, v) = \frac{u \cdot v}{\|u\| \cdot \|v\|}, \qquad \text{(Equation 1)}$$

where u and v represent embedding vectors. Cosine distance ($1 -$ cosine similarity) was used to measure semantic divergence from the initial prompts and between trajectory replicates. This metric is particularly suitable for high-dimensional semantic spaces, as it captures angular relationships independent of vector magnitude.[42]

Since the initial prompt, diversified by the novelty search algorithm, created very elaborate scene descriptions, which are hard to reconstruct from an image, the initial image (step $=$ 0) was skipped when analyzing trajectories.

PCA

To visualize the diversity of image descriptions in low-dimensional space, PCA was applied to a set of prompt embeddings,[43] and if necessary, other embeddings were projected into the same space.

CellPress
OPEN ACCESS

RESOURCE AVAILABILITY

Lead contact

Requests for further information and resources should be directed to and will be fulfilled by the lead contact, Arend Hintze (ahz@du.se).

Materials availability

This study did not generate new unique reagents.

Data and code availability

- All original code to generate images and analyze data and all prompts have been deposited at OSF and are publicly available at https://doi.org/10.17605/OSF.IO/WGDQ4.[44] Raw images generated during the study are not included in that repository due to their large volume but can be regenerated using the provided code. Images for the experiments in which temperature was varied and the final images for each cluster from the experiment in which generators and descriptors were varied can be found at https://doi.org/10.6084/m9.figshare.30553604.[45]
- Analyzed data and summary statistics reported in this paper are publicly available at OSF and can be accessed at https://doi.org/10.17605/OSF.IO/WGDQ4.[44]
- Any additional information required to reanalyze the data reported in this paper is available from the lead contact upon request.

ACKNOWLEDGMENTS

This work was supported in part through computational resources and services provided by the Institute for Cyber-Enabled Research at Michigan State University.

AUTHOR CONTRIBUTIONS

A.H. conceived the study, designed the experiments, and implemented all code. F.P.Å. and J.S. contributed to the methodology, background, discussions on implementation, and interpretation. A.H. wrote the manuscript with input from all authors. All authors reviewed and approved the final version.

DECLARATION OF INTERESTS

The authors declare no competing interests.

DECLARATION OF GENERATIVE AI AND AI-ASSISTED TECHNOLOGIES IN THE WRITING PROCESS

The authors used Claude and ChatGPT to improve grammar, enhance writing clarity, and debug code. They also used these tools to review text sections for comprehensibility, reproducibility, and consistency. After using this tool/service, the authors reviewed and edited the content and take full responsibility for the content of the published article.

SUPPLEMENTAL INFORMATION

Supplemental information can be found online at https://doi.org/10.1016/j.patter.2025.101451.

Received: July 15, 2025
Revised: October 3, 2025
Accepted: November 17, 2025
Published: December 19, 2025

REFERENCES

1. Coppolillo, E., Manzoni, S., Boratto, L., Anelli, V.W., and Di Noia, T. (2025). Algorithmic drift: A simulation framework to study the effects of recommender systems on user preferences. Inf. Process. Manag. *62*, 103676.
2. Mansoury, M., Abdollahpouri, H., Pechenizkiy, M., Mobasher, B., and Burke, R. (2020). Feedback loop and bias amplification in recommender systems. In Proceedings of the 29th ACM international conference on information & knowledge management, pp. 2145–2148.
3. Shumailov, I., Shumaylov, Z., Zhao, Y., Papernot, N., Anderson, R., and Gal, Y. (2024). Ai models collapse when trained on recursively generated data. Nature *631*, 755–759.
4. Shatashvili, I., Shumailov, I., Zhao, Y., Gal, Y., Papernot, N., and Anderson, R. (2023). The curse of recursion: Training on generated data makes models forget. Preprint at arXiv. https://doi.org/10.48550/arXiv.2305.17493.
5. Strogatz, S.H. (2024). Nonlinear Dynamics and Chaos: With Applications to Physics, Biology, Chemistry, and Engineering (Chapman and Hall/CRC).
6. Heusel, M., Ramsauer, H., Unterthiner, T., Nessler, B., and Hochreiter, S. (2017). Gans trained by a two time-scale update rule converge to a local nash equilibrium. In Advances in neural information processing systems, pp. 6626–6637.
7. Taori, R., and Hashimoto, T.B. (2023). Data feedback loops: Model-driven amplification of dataset biases. In International Conference on Machine Learning (PMLR), pp. 33883–33920.
8. Aithal, S.K., Seshadri, S., Karnick, H., and Raj, B. (2024). Understanding hallucinations in diffusion models through mode interpolation. Preprint at arXiv. https://doi.org/10.48550/arXiv.2406.09358.
9. Mosqueira-Rey, E., Hernández-Pereira, E., Alonso-Ríos, D., Bobes-Bascarán, J., and Fernández-Leal, Á. (2023). Human-in-the-loop machine learning: a state of the art. Artif. Intell. Rev. *56*, 3005–3054.
10. Boden, M.A. (2004). The Creative Mind: Myths and Mechanisms (Routledge).
11. Colton, S., and Wiggins, G.A. (2012). Computational creativity: The final frontier? In Proceedings of the 20th European conference on artificial intelligence, pp. 21–26.
12. Soros, L.B., Adams, A.M., Kalonaris, S., Witkowski, O., and Guckelsberger, C. (2024). On creativity and open-endedness. In ALIFE 2024: Proceedings of the 2024 Artificial Life Conference (MIT Press).
13. Wiggins, G.A. (2006). A preliminary framework for description, analysis and comparison of creative systems. Knowl. Base Syst. *19*, 449–458.
14. Oh, C., Song, J., Choi, J., Kim, S., Lee, S., and Suh, B. (2018). I lead, you help but only with enough details: Understanding user experience of co-creation with artificial intelligence. In Proceedings of the 2018 CHI Conference on Human Factors in Computing Systems, pp. 1–13.
15. Agudo, U., Liberal, K.G., Arrese, M., Matute, H., Navarro, C., and Morales, Á. (2024). The impact of ai errors in a human-in-the-loop process. Cogn. Res. Princ. Implic. *9*, 1–12.
16. Doshi, A.R., and Hauser, O.P. (2024). Generative AI enhances individual creativity but reduces the collective diversity of novel content. Sci. Adv. *10*, eadn5290.
17. Ressler, M. (2025). Automated inauthenticity. AI Soc. *40*, 985–994.
18. Runco, M.A. (2023). Updating the standard definition of creativity to account for the artificial creativity of AI. Creat. Res. J. *37*, 1–5.
19. Franceschelli, G., and Musolesi, M. (2025). On the creativity of large language models. AI Soc. *40*, 3785–3795.
20. Rombach, R., Blattmann, A., Lorenz, D., Esser, P., and Ommer, B. (2022). High-resolution image synthesis with latent diffusion models. In Proceedings of the IEEE/CVF conference on computer vision and pattern recognition, pp. 10684–10695.
21. Liu, H., Li, C., Wu, Q., and Lee, Y.J. (2023). Visual instruction tuning. Adv. Neural Inf. Process. Syst. *36*, 34892–34916.
22. Lehman, J., and Stanley, K.O. (2011). Abandoning objectives: evolution through the search for novelty alone. Evol. Comput. *19*, 189–223.
23. Elgammal, A., Liu, B., Elhoseiny, M., and Mazzone, M. (2017). Can: Creative adversarial networks, generating "art" by learning about styles

and deviating from style norms. Preprint at arXiv. https://doi.org/10.48550/arXiv.1706.07068.

24. Zylinska, J. (2020). AI Art: Machine Visions and Warped Dreams (Open Humanities Press).
25. Bender, E.M., Gebru, T., McMillan-Major, A., and Shmitchell, S. (2021). On the dangers of stochastic parrots: Can language models be too big? In Proceedings of the 2021 ACM conference on fairness, accountability, and transparency, pp. 610–623.
26. Birhane, A., Kalluri, P., Card, D., Agnew, W., Dotan, R., and Bao, M. (2022). The values encoded in machine learning research. In Proceedings of the 2022 ACM conference on fairness, accountability, and transparency, pp. 173–184.
27. Crawford, K. (2021). Atlas of AI: Power, Politics, and the Planetary Costs of Artificial Intelligence (Yale University Press).
28. Manovich, L. (2020). AI Aesthetics (Strelka Press).
29. Russell, S. (2019). Human Compatible: Artificial Intelligence and the Problem of Control (Viking).
30. Barocas, S., Hardt, M., and Narayanan, A. (2019). Fairness and machine learning. http://www.fairmlbook.org:fairmlbook.org.
31. Bartlett, F.C. (1932). Remembering: A Study in Experimental and Social Psychology (Cambridge University Press).
32. Kalish, M.L., Griffiths, T.L., and Lewandowsky, S. (2007). Iterated learning: Intergenerational knowledge transmission reveals inductive biases. Psychonomic Bulletin & Review *14*, 288–294.
33. Garrod, S., Fay, N., Lee, J., Oberlander, J., and Macleod, T. (2007). Foundations of representation: Where might graphical symbol systems come from? Cogn. Sci. *31*, 961–987.
34. Griffiths, T.L., and Kalish, M.L. (2007). Language evolution by iterated learning with Bayesian agents. Cogn. Sci. *31*, 441–480.
35. Stanley, K.O., and Miikkulainen, R. (2002). Evolving neural networks through augmenting topologies. Evol. Comput. *10*, 99–127.
36. Tehrani, J.J. (2013). The phylogeny of little red riding hood. PLoS One *8*, e78871.
37. Witzel, E.M. (2012). The Origins of the World's Mythologies (Oxford University Press).
38. Hodgson, D. (2006). Understanding the origins of paleoart: The neurovisual resonance theory and brain functioning. PaleoAnthropology *2006*, 54–67.
39. Mesoudi, A., and Whiten, A. (2004). The hierarchical transformation of event knowledge in human cultural transmission. J. Cognit. Cult. *4*, 1–24.
40. Touvron, H., Martin, L., Stone, K., Albert, P., Almahairi, A., Babaei, Y., Bashlykov, N., Batra, S., Bhargava, P., Bhosale, S., et al. (2023). Llama 2: Open foundation and fine-tuned chat models. Preprint at arXiv. https://doi.org/10.48550/arXiv.2307.09288.
41. Reimers, N., and Gurevych, I. (2019). Sentence-BERT: Sentence embeddings using Siamese BERT-networks. Preprint at arXiv. https://doi.org/10.48550/arXiv.1908.10084.
42. Mikolov, T., Chen, K., Corrado, G., and Dean, J. (2013). Efficient estimation of word representations in vector space. Preprint at arXiv. https://doi.org/10.48550/arXiv.1301.3781.
43. Abdi, H., and Williams, L.J. (2010). Principal component analysis. WIREs Computational Stats. *2*, 433–459.
44. Hintze, A. (2025). Drift and Convergence in Generative AI Leads to Visual Elevator Music (OSF). https://doi.org/10.17605/OSF.IO/WGDQ4.
45. Hintze, A., and Schlossau, J. (2025). Image and description files for Hintze et al emergent motif convergence in iterated language-image generation loops (Patterns 2025). figshare. https://doi.org/10.6084/m9.figshare.30553604.v1.

Patterns, Volume *7*

Supplemental information

Autonomous language-image generation loops converge to generic visual motifs

Arend Hintze, Frida Proschinger Åström, and Jory Schossau

Supplemental Methods 1

October 26, 2025

Converged Image Mosaics After 100 Iterations

The following figures (S1–S16) show the final-step image mosaics from each of the 16 generator–describer model combinations. Each mosaic illustrates the end point of semantic drift, revealing visually similar but subtly model-specific convergences.

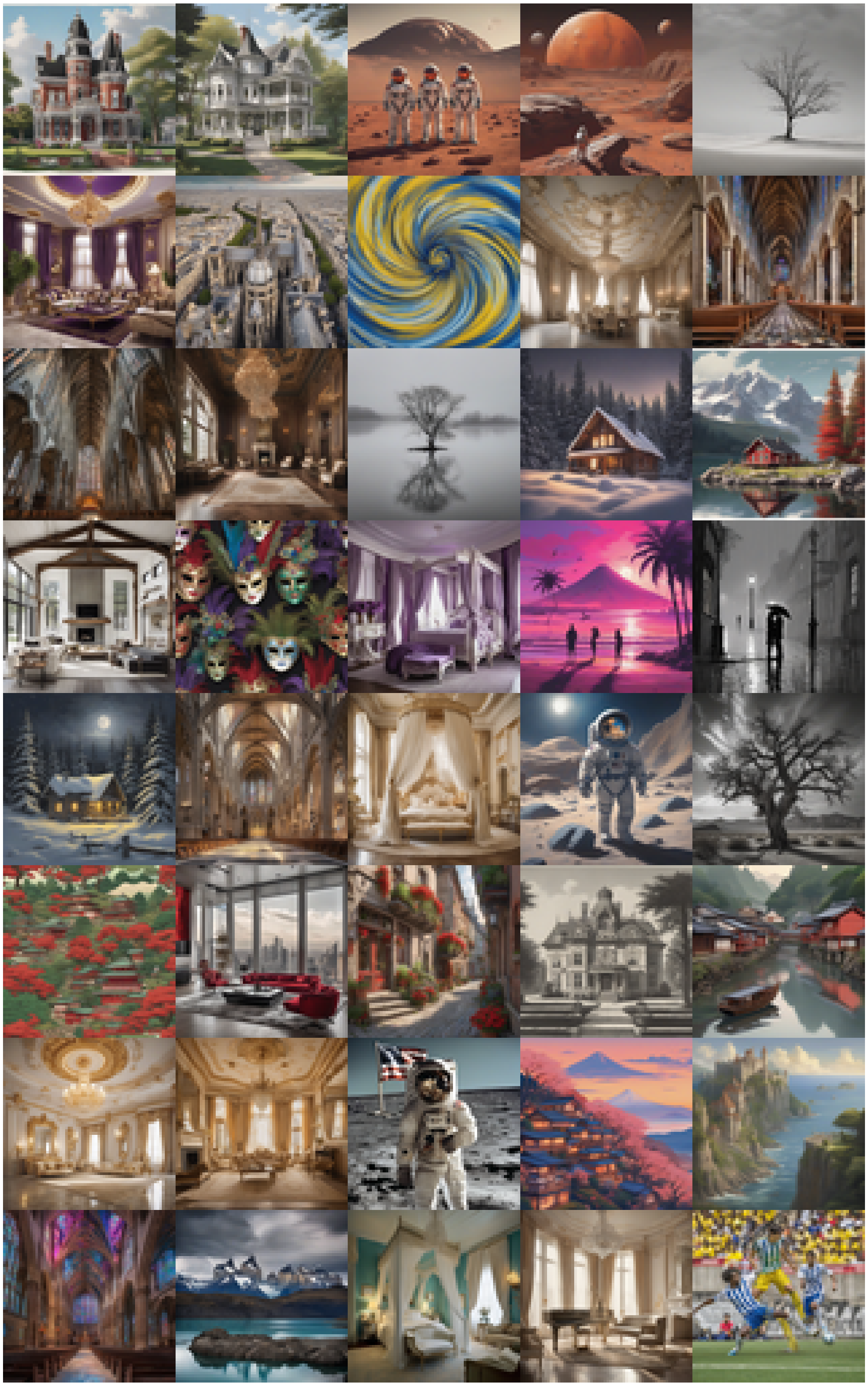

Figure S1: Generator: `stable-diffusion-xl-base-1.0`, Describer: `llava`.

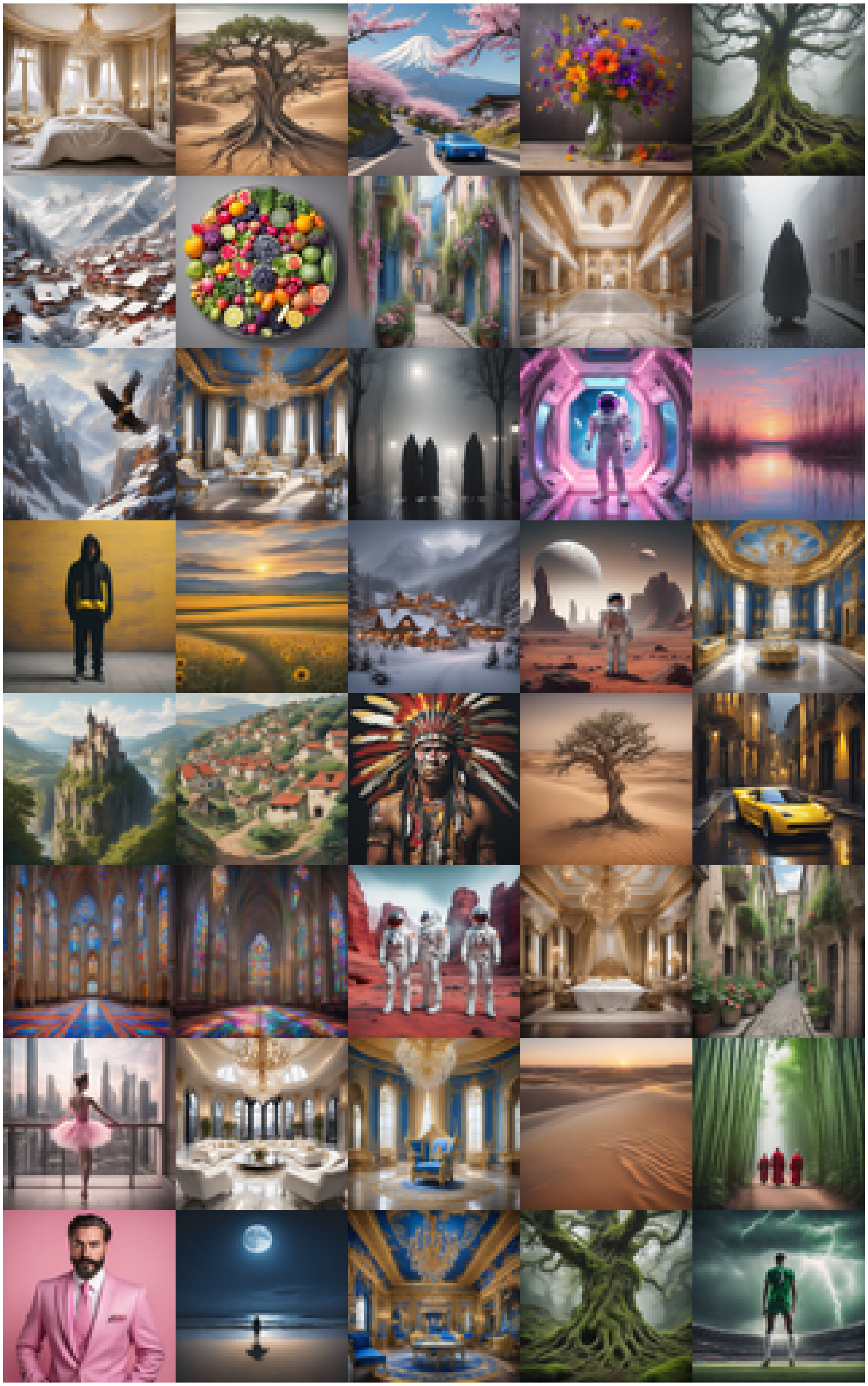

Figure S2: Generator: `segmind-SSD-1B`, Describer: `llava`.

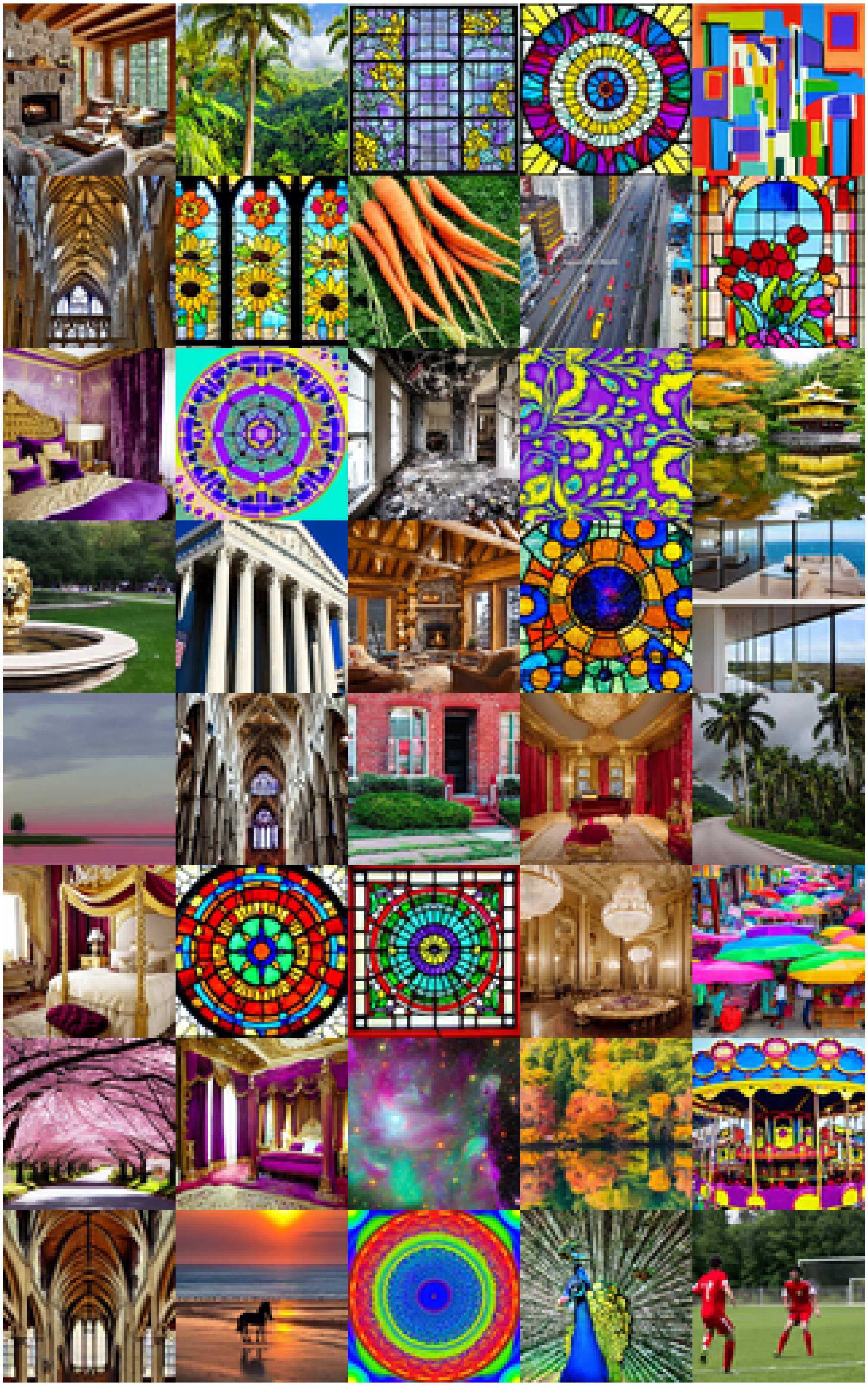

Figure S3: Generator: `stable-diffusion-v1.5`, Describer: `llava`.

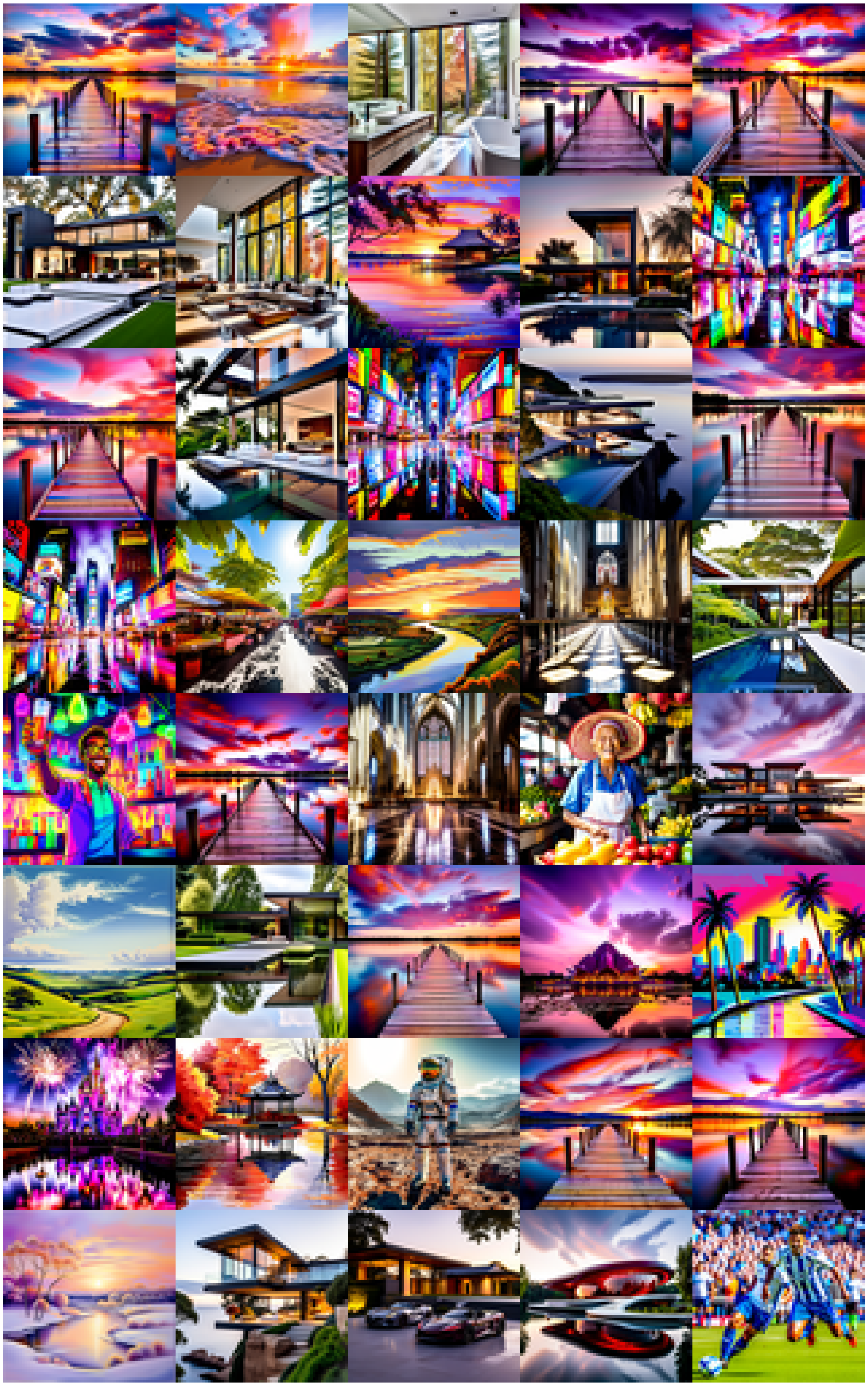

Figure S4: Generator: `playground-v2-aesthetic`, Describer: `llava`.

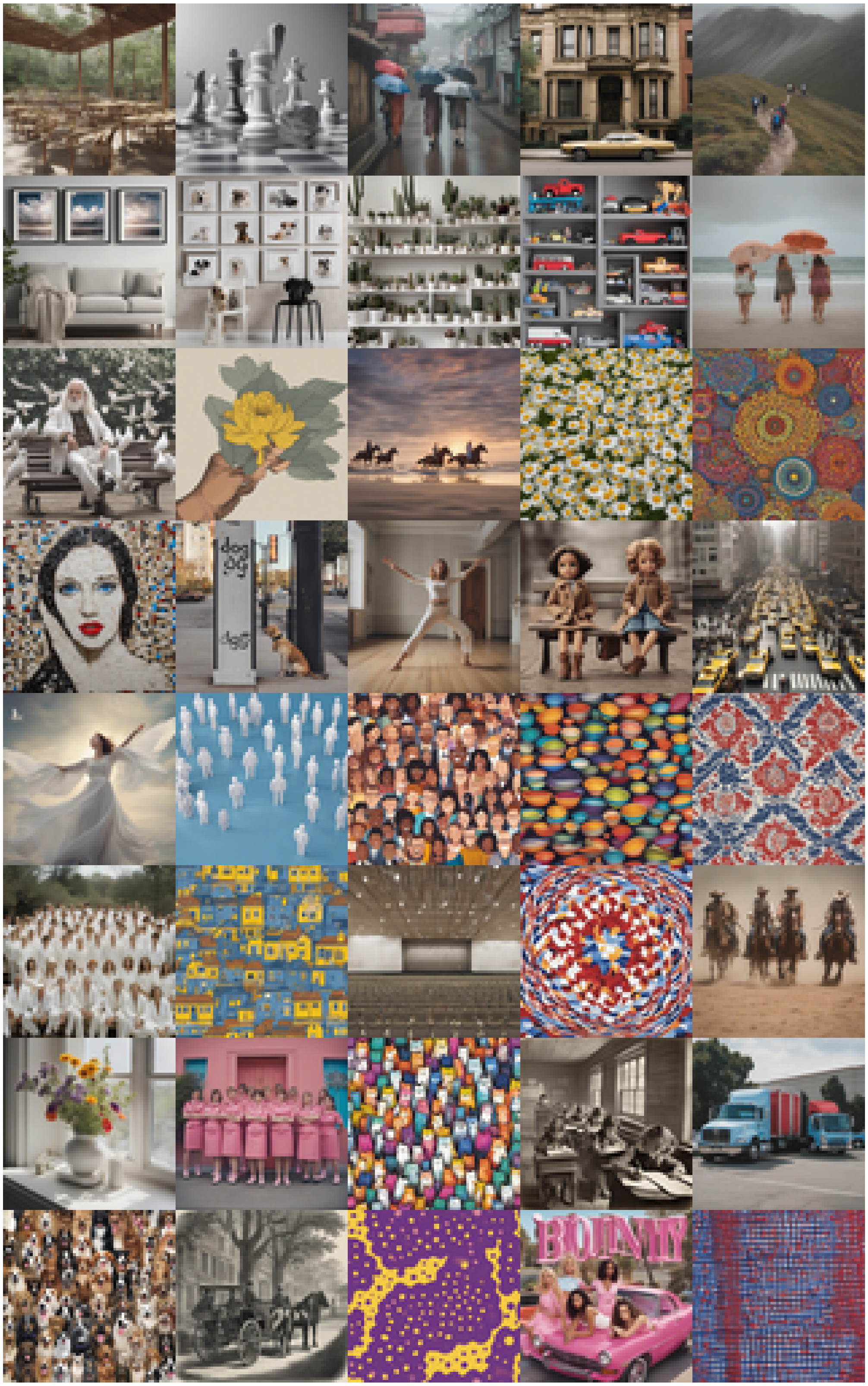

Figure S5: Generator: `stable-diffusion-xl-base-1.0`, Describer: `bakllava`.

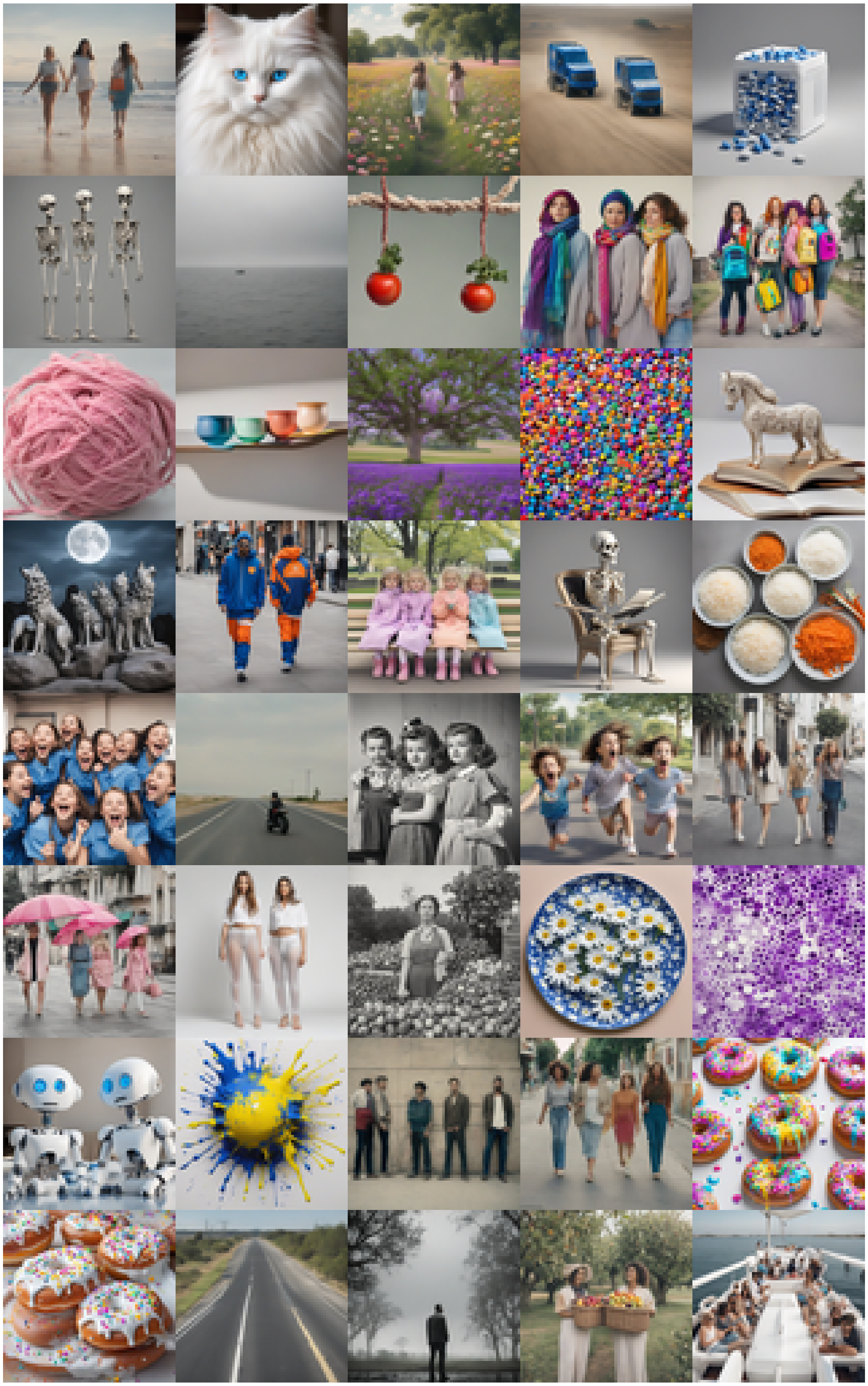

Figure S6: Generator: `segmind-SSD-1B`, Describer: `bakllava`.

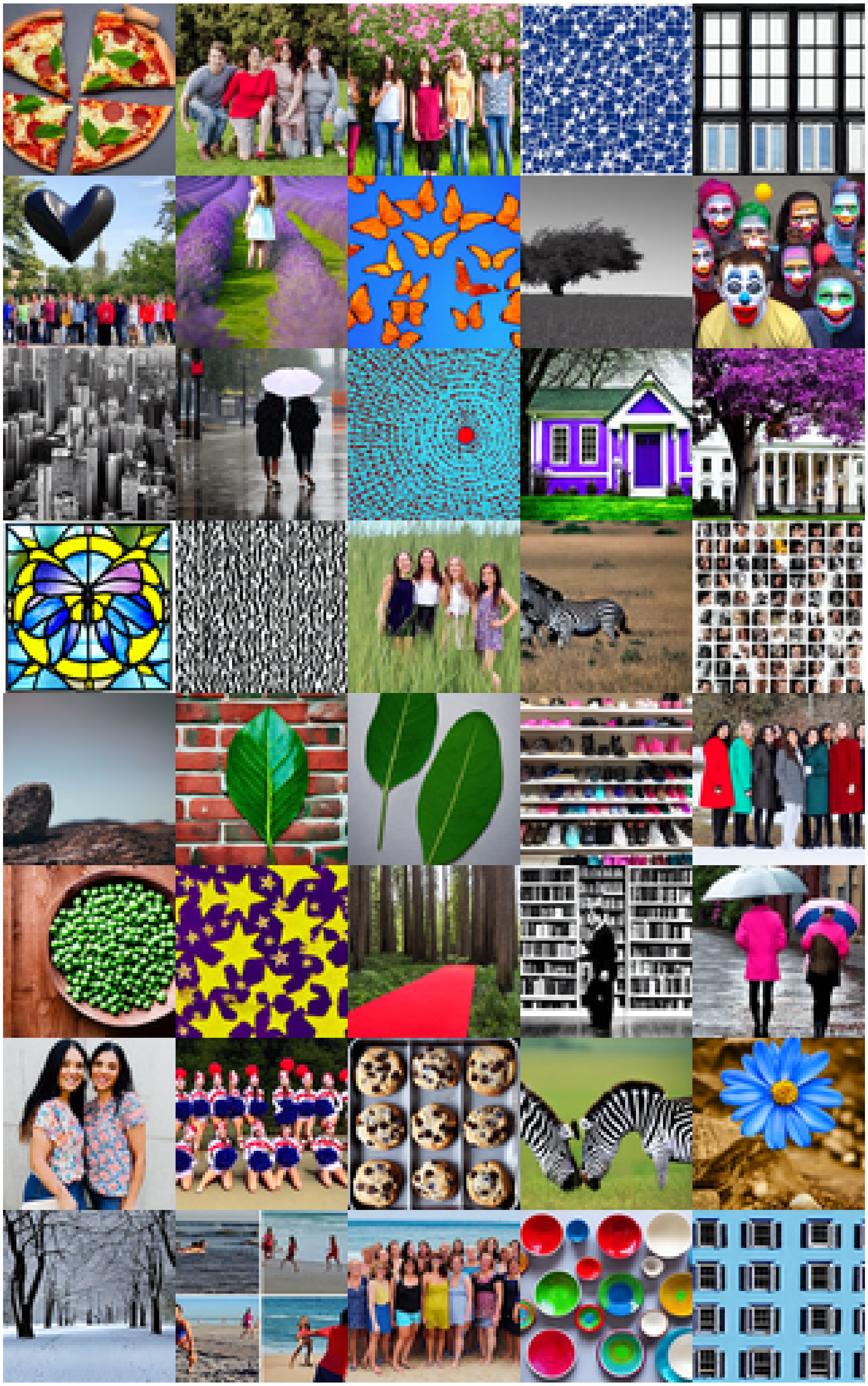

Figure S7: Generator: `stable-diffusion-v1.5`, Describer: `bakllava`.

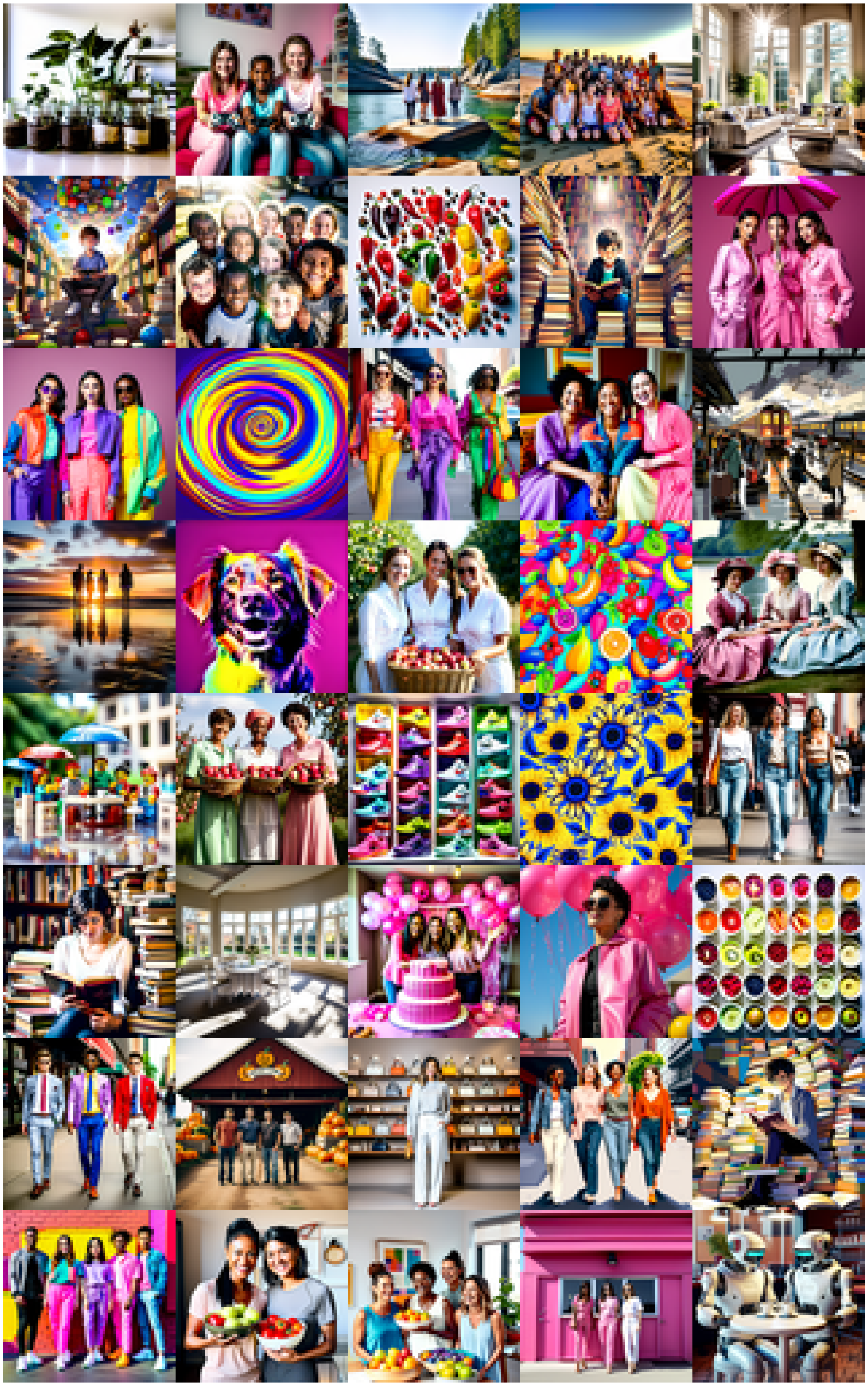

Figure S8: Generator: `playground-v2-aesthetic`, Describer: `bakllava`.

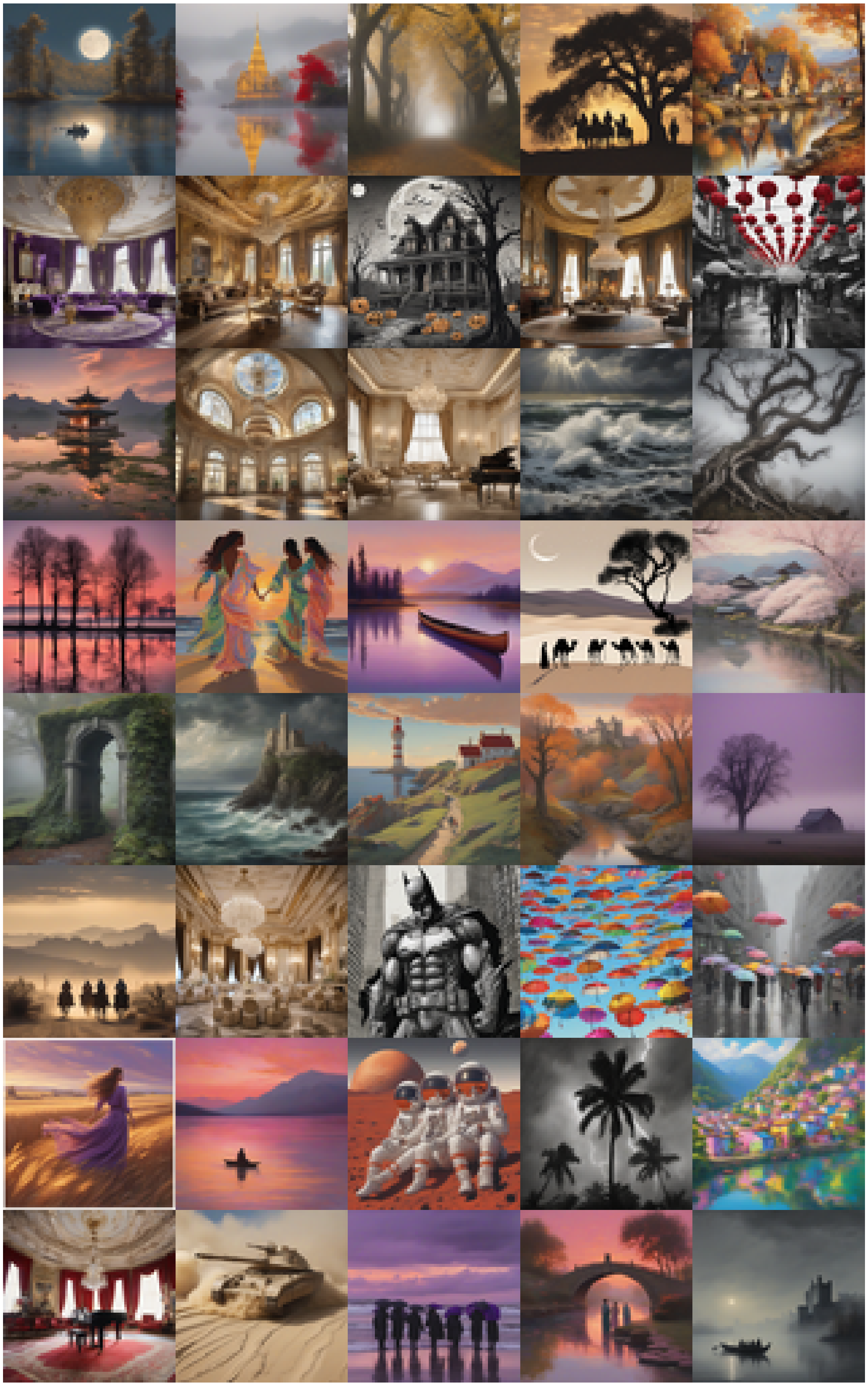

Figure S9: Generator: `stable-diffusion-xl-base-1.0`, Describer: `llava:13b-v1.6`.

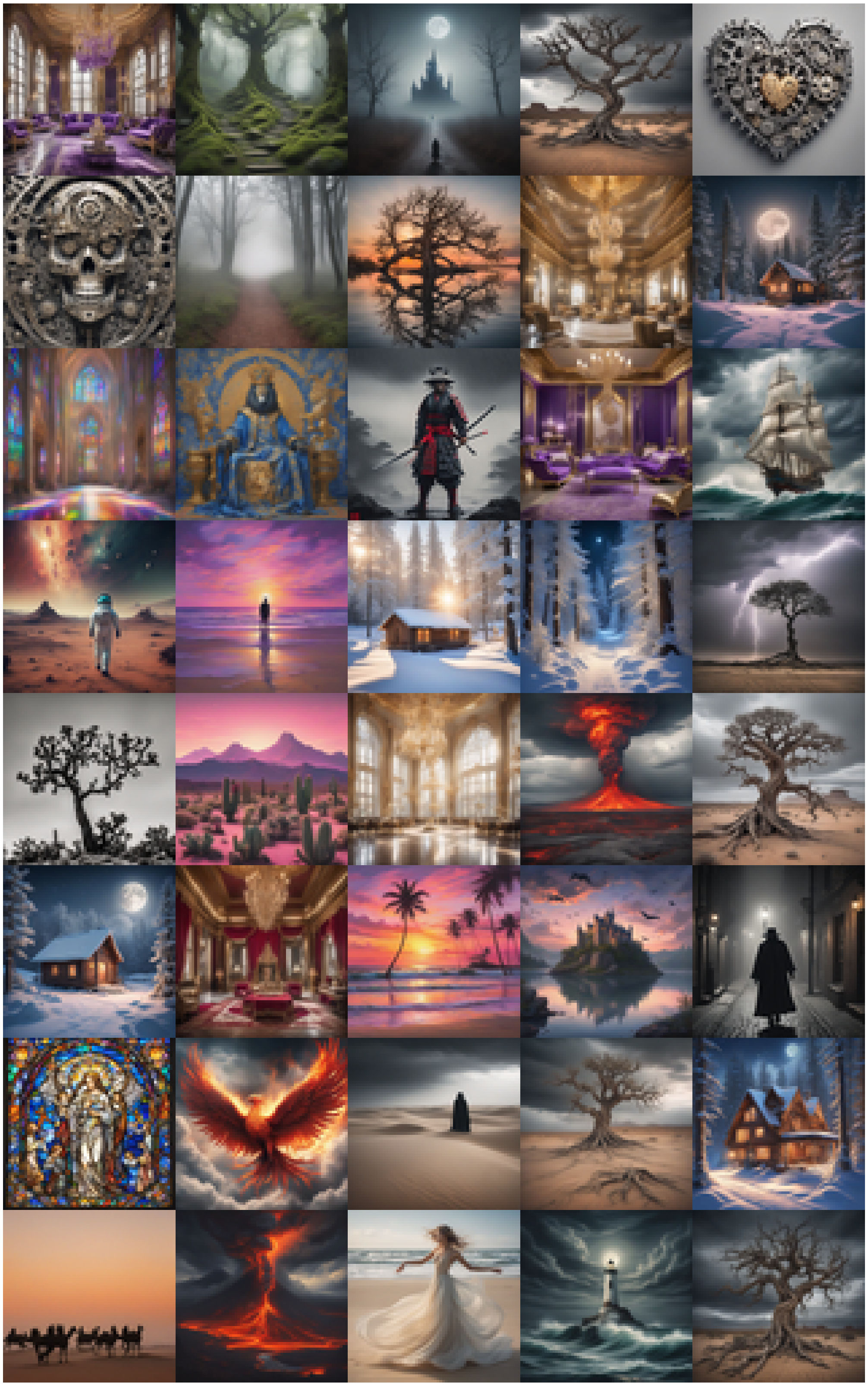

Figure S10: Generator: `segmind-SSD-1B`, Describer: `llava:13b-v1.6`.

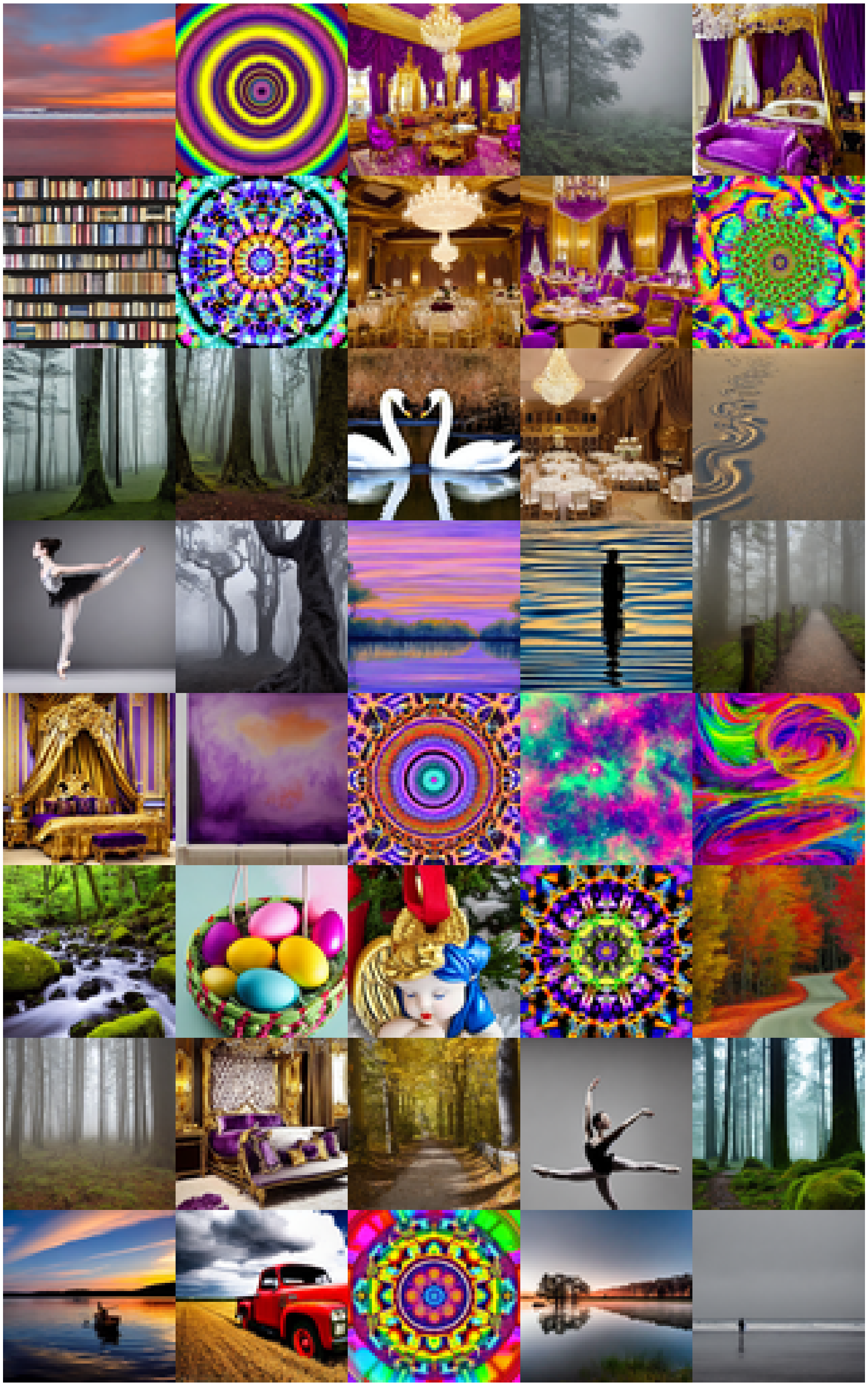

Figure S11: Generator: `stable-diffusion-v1.5`, Describer: `llava:13b-v1.6`.

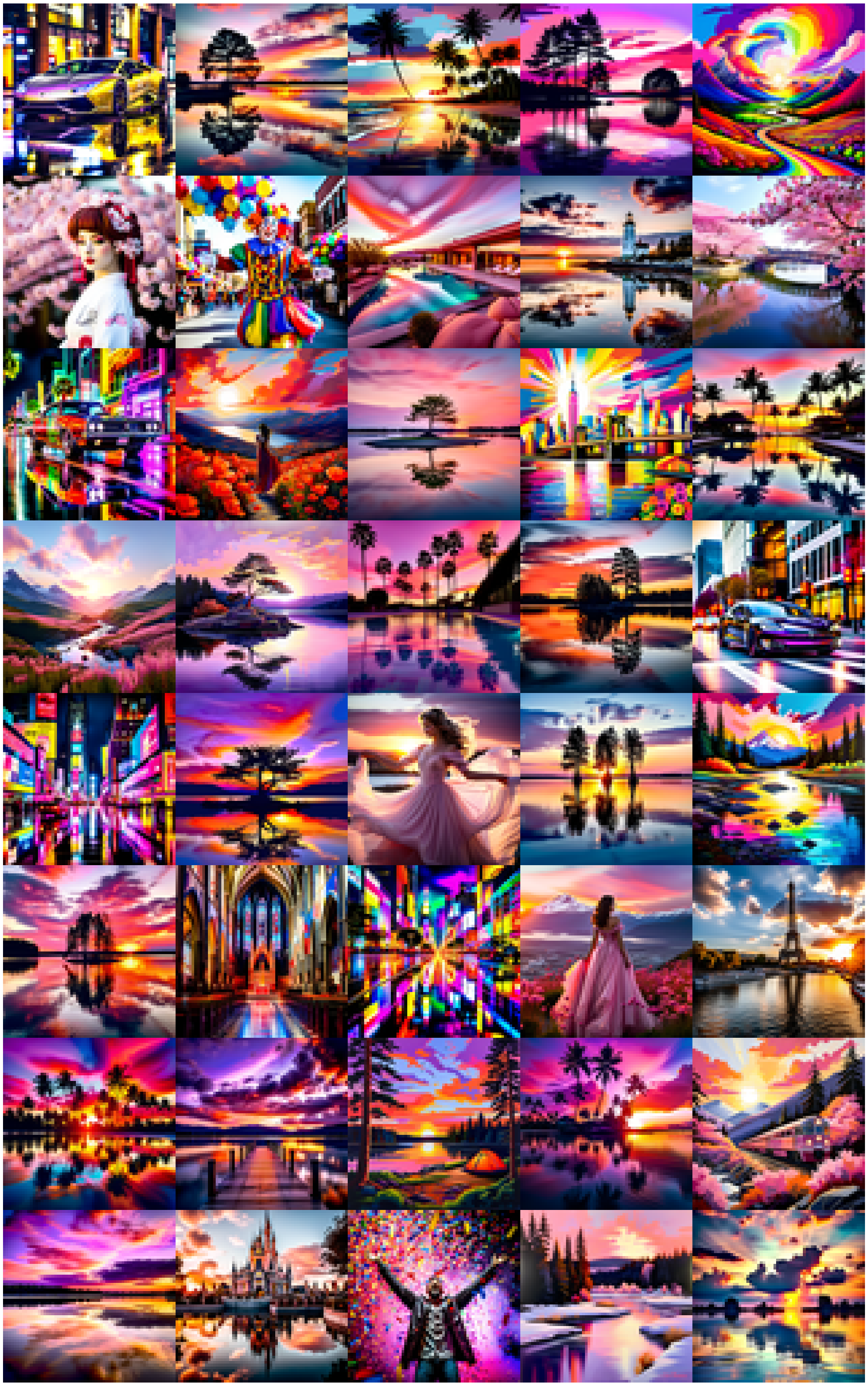

Figure S12: Generator: `playground-v2-aesthetic`, Describer: `llava:13b-v1.6`.

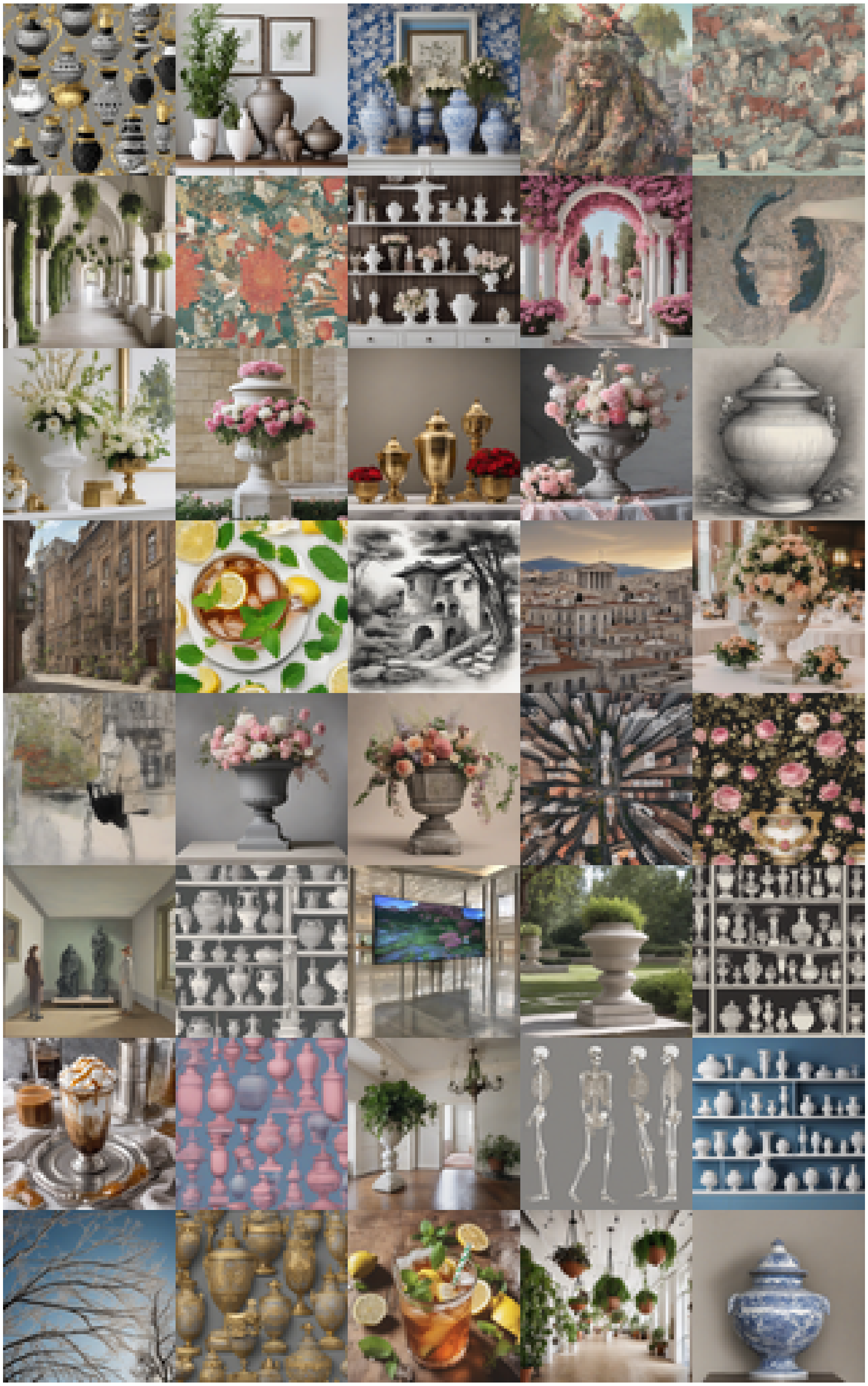

Figure S13: Generator: `stable-diffusion-xl-base-1.0`, Describer: `moondream`.

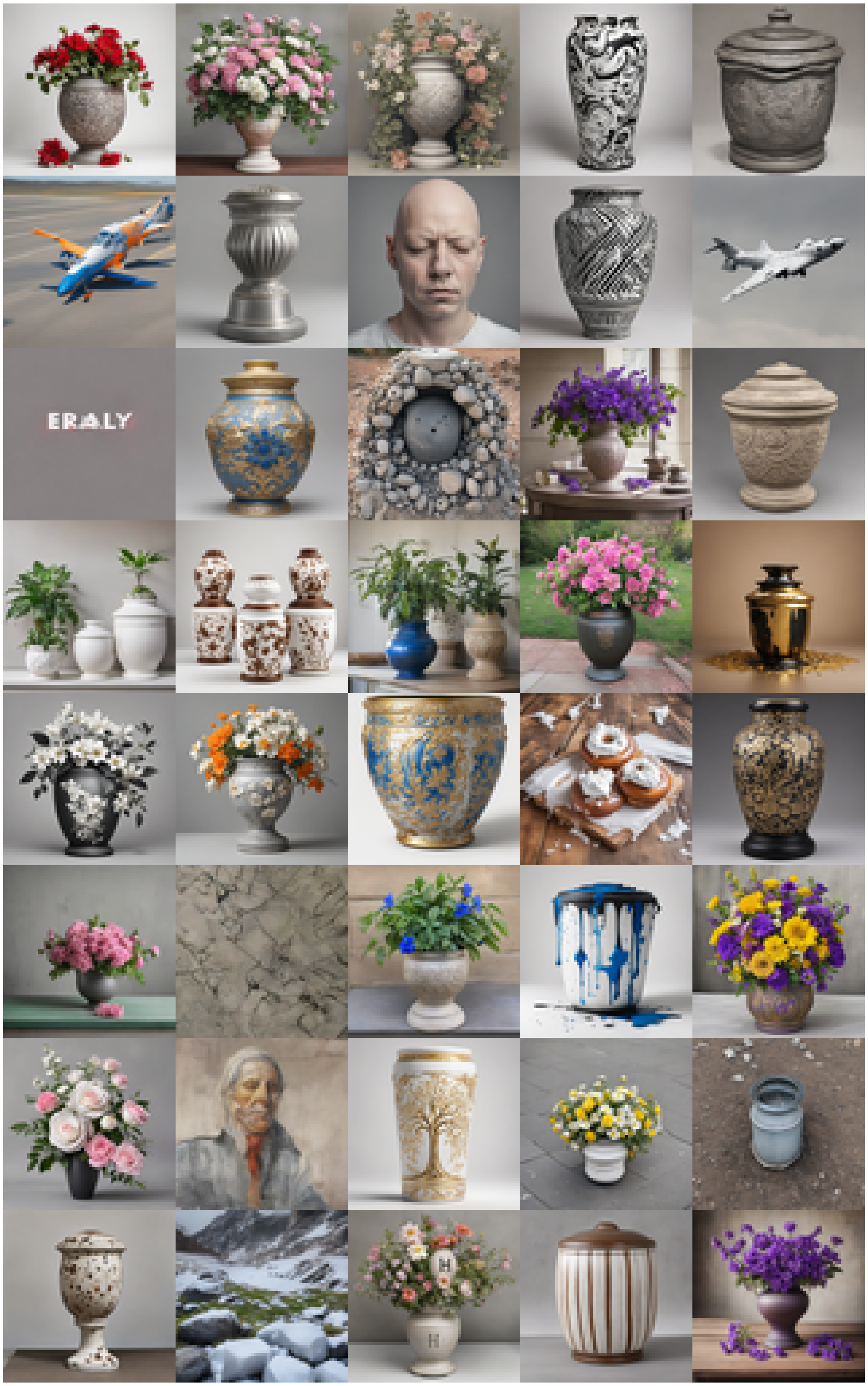

Figure S14: Generator: `segmind-SSD-1B`, Describer: `moondream`.

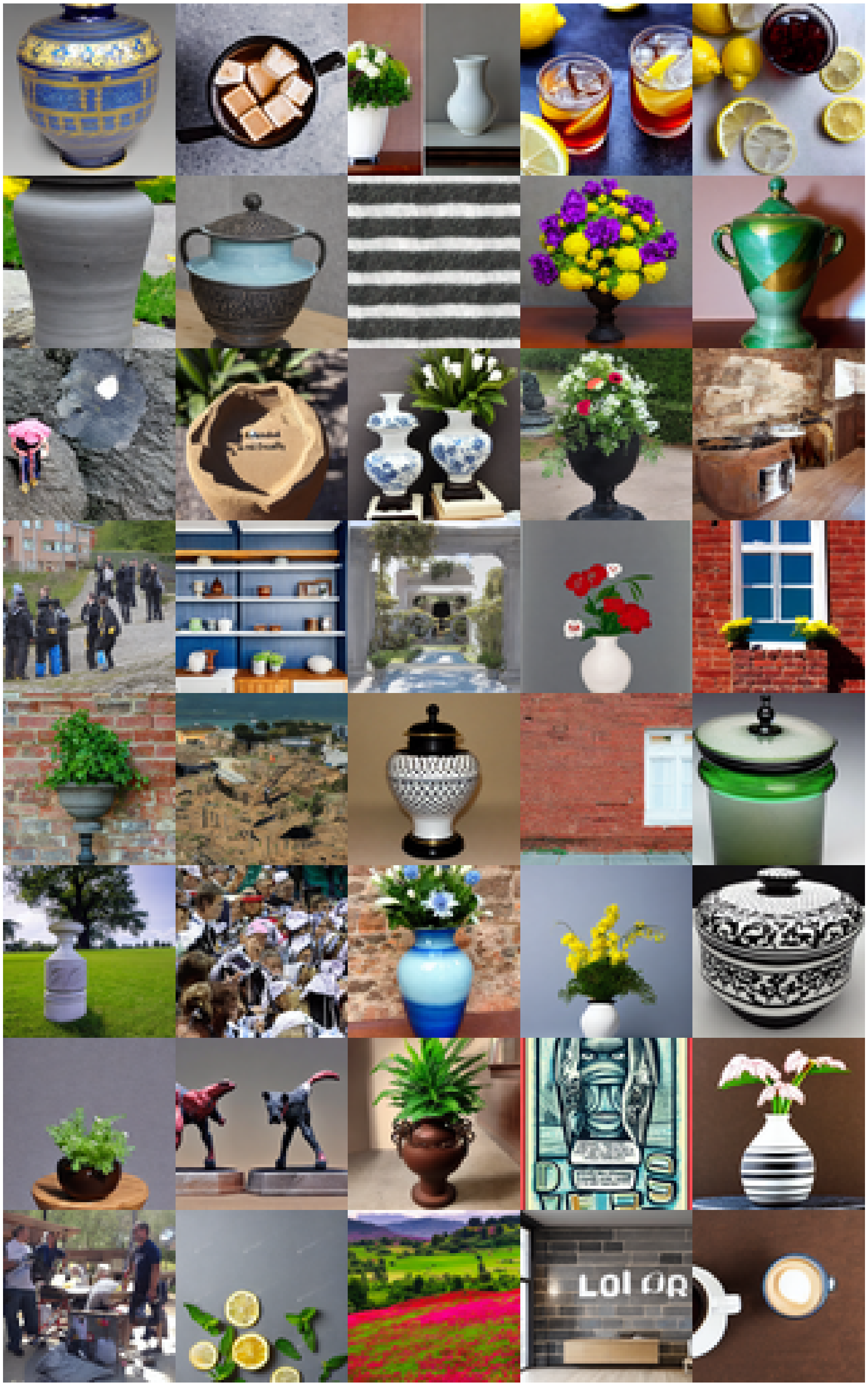

Figure S15: Generator: `stable-diffusion-v1.5`, Describer: `moondream`.

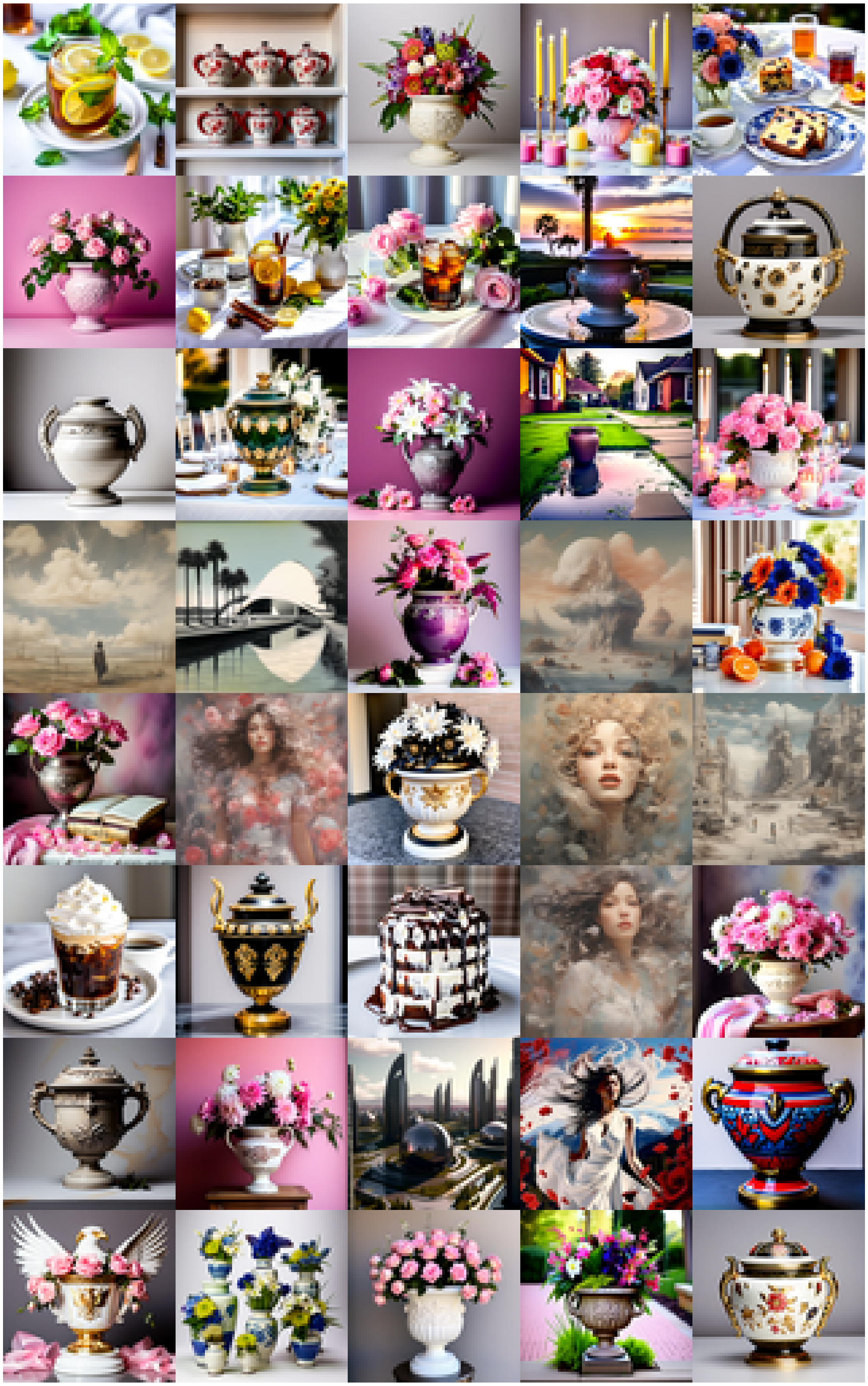

Figure S16: Generator: `playground-v2-aesthetic`, Describer: `moondream`.

Supplemental Methods 2

October 26, 2025

Long-term Behavior Example

While we observed convergence over the 100 steps we performed for all the experiments shown before, one might ask about the long-term behavior. Are images stable, or do they keep changing, and if they change, do they just move from one possible attractor to the next, or are they exploring new areas?

While interesting to explore in the future, the term "long" is rather ambiguous. Here, we considered 100 rounds, but what is a realistic number in the real world? So far we have seen a few rounds of improvement reaching up to gpt-5, and as such, whatever content produced by LLM might have gone into the training set at most 4 times in the case of OpenAI. Our experiment, however, is not concerned with learning from AI-generated content, but possible recreation and re-interpretation loops, as they might happen within agentic systems. For us, it is hard to guess a realistic number, but we think that 100 rounds is already excessively high. Nevertheless, we superficially explored the long-term behavior in experiments that ran for 1000 rounds.

We observe the known attractors to reappear, and images being stable over very long periods (several 100 rounds), but to also, occasionally, to move from one attractor to the next (for an example see Figure S17). These initial impressions propose a new set of interesting aspects to explore: How stable are images and prompts? Are some attractors more attractive than others? Is there a preference for this process, suggesting that some attractors are more likely to follow others?

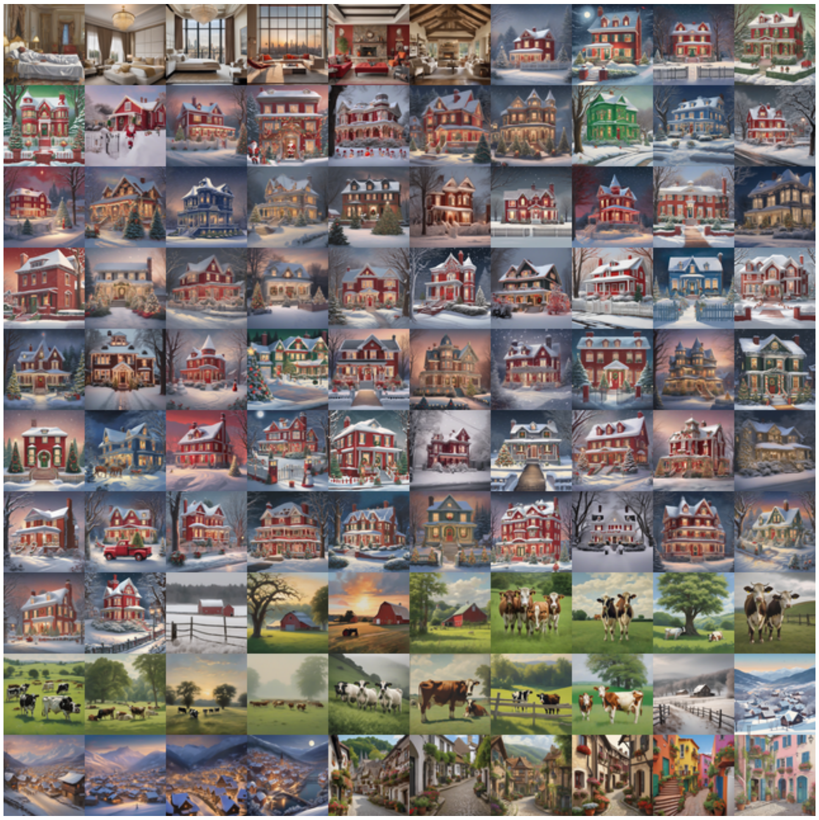

Figure S17: Long-term behavior of a prompt-image generation loop, run for 1000 rounds. Every tenth image is shown.
